# Exploring Immune Cell Diversity in the Lacrimal Glands of Healthy Mice: A Single-Cell RNA-Sequencing Atlas

**DOI:** 10.3390/ijms25021208

**Published:** 2024-01-19

**Authors:** Qiwei Fan, Ruyu Yan, Yan Li, Liyuan Lu, Jiangman Liu, Senmao Li, Ting Fu, Yunxia Xue, Jun Liu, Zhijie Li

**Affiliations:** 1 Department of Pathology, School of Medicine, Jinan University, Guangzhou 510632, China; fanqw@stu2022.jnu.edu.cn (Q.F.); jiangman@stu2023.jnu.edu.cn (J.L.); 2 International Ocular Surface Research Center, Key Laboratory for Regenerative Medicine, Institute of Ophthalmology, Jinan University, Guangzhou 510632, China; rluves@stu2022.jnu.edu.cn (R.Y.); liyan9715@163.com (Y.L.); luliyuan@stu2022.jnu.edu.cn (L.L.); medlisenmao@gmail.com (S.L.); tfuting@jnu.edu.cn (T.F.); xueyx@jnu.edu.cn (Y.X.); junliu@jnu.edu.cn (J.L.); 3 Department of Ophthalmology, The First Affiliated Hospital of Jinan University, Jinan University, Guangzhou 510630, China

**Keywords:** mouse, extraorbital lacrimal gland, single-cell sequencing, transcriptomics, immune cells

## Abstract

The lacrimal gland is responsible for maintaining the health of the ocular surface through the production of tears. However, our understanding of the immune system within the lacrimal gland is currently limited. Therefore, in this study, we utilized single-cell RNA sequencing and bioinformatic analysis to identify and analyze immune cells and molecules present in the lacrimal glands of normal mice. A total of 34,891 cells were obtained from the lacrimal glands of mice and classified into 18 distinct cell clusters using Seurat clustering. Within these cell populations, 26 different immune cell subpopulations were identified, including T cells, innate lymphocytes, macrophages, mast cells, dendritic cells, and B cells. Network analysis revealed complex cell-cell interactions between these immune cells, with particularly significant interactions observed among T cells, macrophages, plasma cells, and dendritic cells. Interestingly, T cells were found to be the main source of ligands for the Thy1 signaling pathway, while M2 macrophages were identified as the primary target of this pathway. Moreover, some of these immune cells were validated using immunohistological techniques. Collectively, these findings highlight the abundance and interactions of immune cells and provide valuable insights into the complexity of the lacrimal gland immune system and its relevance to associated diseases.

## 1. Introduction

The lacrimal gland, located in the human orbital cavity, is a versatile exocrine gland that plays a central role in maintaining the physiological balance of the eye [1]. The primary secretory function of the lacrimal gland is the production of tears, which serve as a lubricant for the ocular surface, protecting it from desiccation and abrasion caused by friction with the eyelids [2]. Tears contain a variety of antimicrobial components, including antimicrobial peptides, immunoglobulin A (IgA), and complement components [3]. These molecular fortifications, complemented by the mechanical action of the blink, collectively act as sentinels to protect the ocular surface from microbial invasion. In addition, tears contain a wealth of essential nutrients that promote the proliferation and differentiation of ocular surface cells [4]. Notably, tears are also recognized as a physiological response to emotional stimuli, such as grief, joy, or fear, and serve as a conduit for emotional expression [5]. Thus, a thorough understanding of the cellular makeup of the lacrimal gland is paramount to unraveling its physiological functions.

Like other exocrine glands, the lacrimal gland harbors a diverse repertoire of immune cells, each contributing to its normal function through a spectrum of mechanisms [6]. Equipped with an arsenal of immune defense, pathogen recognition, and resistance to microbial invasion, these immune sentinels provide a bulwark against microbial invasion. In addition, these immune cells participate in tissue regeneration and repair by orchestrating the elimination of senescent, damaged, or deceased cells and cellular debris, thereby facilitating tissue homeostasis. The immunological orchestra is further enriched by the secretion of immunoregulatory factors, such as cytokines and antibodies, which orchestrate immune responses and avert the dangers of excessive activation or immunological dysfunction. In addition, immune cells orchestrate a symphony of metabolic regulators that actively participate in the orchestration of energy metabolism and nutrient homeostasis within the lacrimal gland.

The lacrimal gland contains a variety of immune cell subpopulations [6,7], including B lymphocytes [8], T lymphocytes [7], natural killer (NK) cells [8], γδ T cells [7], macrophages (MΦ) [9,10], and dendritic cells (DCs) [11,12], each with its own unique mechanism of physiological regulation. B lymphocytes, which specialize in antibody production, play a central role in the exocrine glands, particularly in the production of mucosal IgA. Meanwhile, T lymphocytes, characterized by their exquisite specificity, spearhead the recognition and elimination of invading pathogens within the exocrine glands. Notably, NK cells have the remarkable ability to destroy pathogens and tumor cells independently of specific recognition, primarily clearing the lacrimal gland of invading pathogens and cellular intruders. Finally, DCs, acting as master antigen-presenters, capture and present invading pathogens and antigens, thereby recruiting other immune cohorts within the lacrimal gland, as exemplified by the activation of T lymphocytes.

Recently, single-cell RNA sequencing (scRNA-seq) technology has provided unprecedented insights into cellular heterogeneity, enabling the identification of unexpected cell characteristics. In particular, the construction of cellular atlases for normal tissues based on scRNA-seq data lays the foundation for the analysis of cells in pathological states, leading to a deeper understanding of the pathological mechanisms underlying the lacrimal gland. While some recent scRNA-seq studies have focused on the analysis of secretory cells within glandular acini [13,14,15], our knowledge of immune cells within the stromal layer of the mouse lacrimal gland remains limited. Therefore, the aim of our study was to further analyze the immune cell populations within the lacrimal gland, along with their characteristics, at the resolution offered by scRNA-seq and thereby gain deeper insights into the diversity of immune cells within the lacrimal gland.

## 2. Results

### 2.1. Single-Cell Transcription of Immune Cell Subpopulations in Mouse ELGs

We collected four fresh ELG samples from 12–14-week-old male C57/B6J mice, pooling bilateral lacrimal glands from the same mice into one sample to ensure an adequate number of cells was collected. The collected samples were then subjected to scRNA-seq using the 10× Genomics platform (Figure S1A). To ensure data quality, we implemented quality control measures to remove double/multiple cells and apoptotic cells (Figure S1B,C). This resulted in a total of 34,891 mouse ELG cell samples for further analysis.

Using Seurat, a graph-based cluster analysis tool, we performed unsupervised clustering and UMAP dimensionality reduction analysis on the collected cell samples; this allowed us to identify 18 transcriptionally defined cell subpopulations (Figure S1D). To determine the general location of each subpopulation, we utilized classical marker genes and UMAP visualization (Figure S1D).

**Figure 1:**
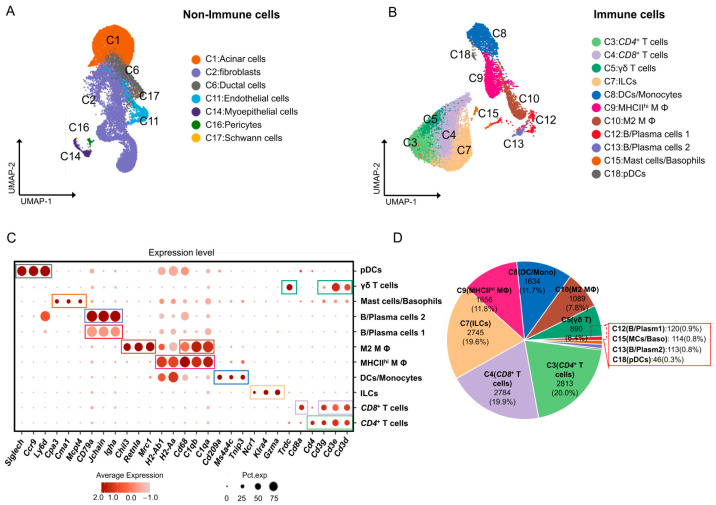
scRNA-seq of immune cells in mouse ELGs. (**A**,**B**) UMAP plot of scRNA-seq data of nonimmune cells clustered independently (**A**) and immune cells clustered independently from four biological replicates (**B**). Colors represent cell types. (**C**) Dot plot showing the expression of selected cell-type-specific marker genes. The dot size denotes the percentage of cells expressing the marker gene in the respective cell population, and the dot color denotes the natural logarithm of normalized RNA expression. Different colored boxes represent different cell clusters. (**D**) Pie chart showing the number of cells in each subpopulation and their respective percentages.

To categorize the cells based on gene expression, we focused on the leukocyte common antigen gene *Ptprc*. By doing so, we were able to classify the cells into two main categories: nonimmune and immune cells (Figure 1). Within the nonimmune cell category, we identified several distinct subpopulations, including epithelial cells characterized by the expression of *Espam* and *Fxyd3* [13,16]; myoepithelial cells characterized by the expression of *Acta2* and *Krt14* [17]; vascular endothelial cells characterized by the expression of *Cyyr1*, *Esam*, and *Flt1* [18]; fibroblasts characterized by the expression of *Col1a1*, *Gsn*, and *Apod* [19]; pericytes characterized by the expression of *Kcnj8*, *Vtn*, and *Abcc9* [20]; and Schwann cells characterized by the expression of *Foxd3*, *Plp1*, and *Kcna1* [21] (Figure S1C–E). Importantly, we identified seven immune cell subpopulations in adult male mouse ELGs, each with well-defined characteristics [22]. These subpopulations included T cells expressing *Cd3d*, *Cd3e*, and *Cd3g* [23], including *CD4*^+^ T cells, *CD8*^+^ T cells, and γδ T cells with high expression of *Trdc* [24,25,26], innate lymphoid cells (ILCs) expressing *Klra4*, *Ncr1*, and *Gzma* [27,28,29], macrophages (MΦ) expressing *C1qa*, *C1qb*, and *CD68* [30,31], including MHC II^hi^ MΦ with high expression of MHC class II molecules (*H2-Ab1*, *H2Aa*) and M2 MΦ with high expression of *Mrc1* (encoding CD206), *Retnlα* (*Fizz1)*, and *Chil3* (*Chi3l3*, *Ym1*) [32], DCs expressing *Cd209a*, *Ms4a4c*, and *Tnip3* [33,34], plasmacytoid dendritic cells (pDCs) expressing *Siglech*, *Ccr9*, and *Ly6d* [35,36,37], B cells and plasma cells expressing *Igha*, *Jchain*, and *Igkc* [19,38], and mast cells expressing *Mcpt4*, *Cma1*, and *Cpa3* [39,40,41] (Figure 1C).

To determine the distribution of these immune cell subpopulations, we calculated their number and percentage in the total immune cell population of the ELGs. Our results showed that the T-cell subpopulation had the highest percentage, accounting for 46.1% of the total cell population, followed by ILCs (18.3%), MΦs (18.4%), and DCs (11.1%) (Figure 1D). In conclusion, our scRNA-seq analysis revealed distinct immune cell subpopulations in adult male mouse ELGs. Further investigation of these subpopulations may provide valuable insights into their roles and functions in the immune response within ELGs.

### 2.2. T Cells in Mouse ELGs

**Figure 2:**
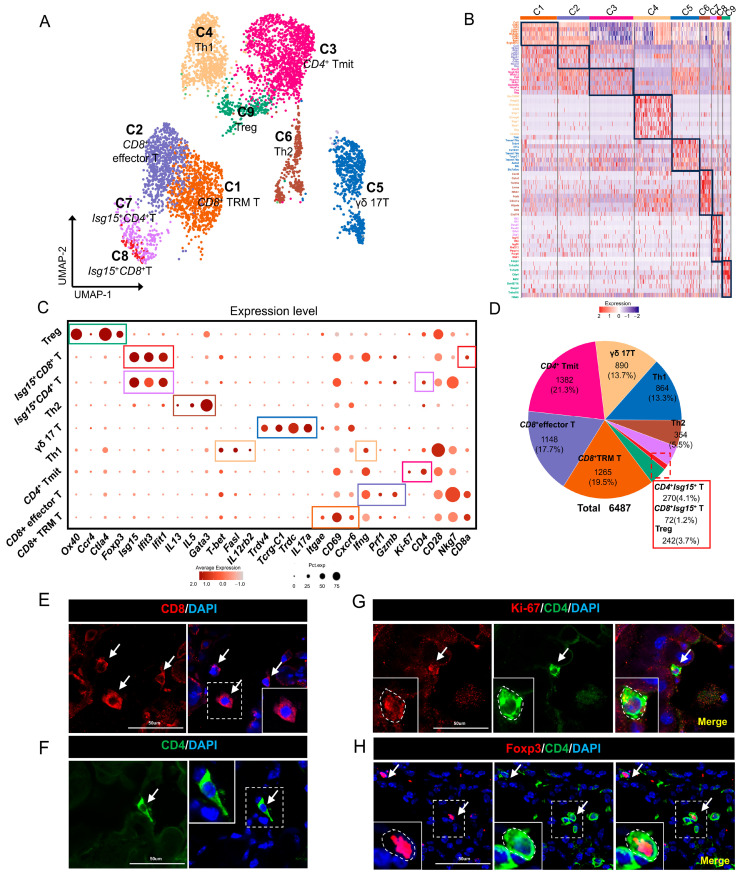
T-cell subsets in mouse lacrimal glands. (**A**) UMAP visualization of T-cell subsets in mouse ELGs. (**B**) Heatmap of Z scores for scaled expression values of DEGs for each cluster. Colors are based on the natural logarithm of normalized RNA expression. The black boxes indicate the DEGs within each cluster. (**C**) Dot plot showing marker genes in each cluster of cells. The dot size denotes the percentage of cells expressing the marker gene in the respective cell population, and the dot color denotes the natural logarithm of normalized RNA expression. Different colored boxes represent different cell clusters. (**D**) Pie chart showing the number of cells in each subpopulation of T cells and their respective percentage of the total T-cell population. (**E**) Histological immunofluorescence staining of the lacrimal gland showing anti-CD8 labeled cells, white arrows point to positively stained cells, scale bar = 50 µm. (**F**) Histological immunofluorescence staining of the lacrimal gland showing anti-CD4-labeled cells, white arrows point to positively stained cells, scale bar = 50 µm. (**G**) Histological immunofluorescence staining of the lacrimal gland showing cells costained with PE-conjugated anti-Ki-67 and FITC-conjugated anti-CD4 costained cells, white arrows point to positively stained cells, scale bar = 50 µm. (**H**) Histological immunofluorescence staining of the lacrimal gland for PE-conjugated anti-Foxp3 and FITC-conjugated anti-CD4 antibody costained cells, white arrows point to positively stained cells, scale bar = 50 µm.

To investigate the characteristics of T-cell subpopulations in the mouse lacrimal glands, we further analyzed 6487 T cells selected from four samples using UMAP dimensional reduction analysis after a second round of clustering analysis (Figure 2A,B).

Among the cell clusters, Cluster 1 was identified as *CD8*^+^ tissue-resident memory (TRM) T cells due to its high expression of *CD8a*, *CD28*, *CD69*, *Cxcr6*, and *Itgae* [42]. In contrast to Cluster 1, Cluster 2 represented a distinct group of CD8-positive T cells. These cells were classified as *CD8*^+^ effector T cells based on their high expression of effector T-cell marker genes such as *Gzmb*, *Prf1*, and *Ifng* [43] (Figure 2C). Further analysis of Cluster 2 cells revealed elevated levels of the cytotoxicity-related genes *Nkg7*, *Gzmk*, and *Ctla2a*, supporting their annotation as *CD8*^+^ cytotoxic T cells [44,45].

Immunofluorescence validation confirmed the presence of *CD8*^+^ T cells in the lacrimal glands (Figure 2E). Figure S2 displays the functional KEGG pathways and GO-BP analysis of gene expression in *CD8*^+^ TRM T cells and *CD8*^+^ effector T cells. Cluster 3 was characterized by the expression of *CD4* and the proliferation-related gene *Mki67* (encoding Ki-67) and was identified as proliferating *CD4*^+^ T_mit cells [46] (Figure 2C). Immunofluorescence analysis further confirmed the presence of *Mki67*^+^*CD4*^+^ T_mit cells (Figure 2G). Cluster 4, in addition to expressing *CD4*, also specifically expressed *IL12rb2*, *T-bet* and *FasL*, suggesting that these cells are Th1 cells [47,48,49]. Cluster 5 expressed *Trdc* (encoding T-cell receptor δ constant chain), *Il17a*, and *Tcrg-C1* (encoding T-cell receptor γ constant chain-1) [26,50,51]. Additionally, this group of cells only expressed the variable region gene *Trdv4* of the T-cell receptor (TCR) γ chain. Therefore, Cluster 5 was annotated as IL-17a-producing Vγ4 γδ T cells (Figure 2C). Functional KEGG and GO-BP analyses showed that genes expressed by the IL-17a-producing Vγ4 γδ T-cell population were mainly enriched in IL-17A, protein export, and Fc gamma R-mediated phagocytosis and were relatively abundant in biological processes such as response to oxygen-containing compound, response to endogenous stimulus, and cell activation involved in immune response (Figure S2). Cluster 6, while expressing *CD4*, also showed high expression of the Th2-specific markers *Gata3*, *IL5*, and *IL13* [52,53,54]. Therefore, Cluster 6 was identified as Th2 cells. Cluster 7, while expressing *CD8*, also demonstrated significant upregulation of the *Ifit1*, *Ifit2*, *Isg15*, and *Isg20* genes. Therefore, this specific T-cell population was characterized as *Isg15*^+^ *CD8*^+^ T cells [55,56,57] (Figure 2C). In contrast, Cluster 8 exhibited high expression of the interferon (IFN)-related genes *Ifit1*, *Ifit2*, *Isg15*, and *Isg20*, but unlike Cluster 7, these cells were *CD4* positive; consequently, they were classified as *Isg15*^+^ *CD4*^+^ T cells.

Finally, Cluster 9 showed high expression of *CD4*, *Foxp3*, *Ccr4*, and *Ox40* and was classified as regulatory T cells (Tregs) [58,59,60,61]. In addition, Tregs in the lacrimal glands also showed high expression of the marker genes *Ctla4* (encoding cytotoxic T lymphocyte-associated protein 4) and *Nrp1* (encoding neuropilin-1), one of the receptors of semaphorin3A associated with Treg immunosuppressive function. Immunofluorescence analysis confirmed the presence of *CD4*^+^*Foxp3*^+^ Tregs in the lacrimal glands (Figure 2H). Functional KEGG and GO-BP analyses showed that genes expressed by the Treg cell population were mainly enriched in the T-cell receptor signaling pathway, Jak-STAT signaling pathway, and chemokine signaling pathway and were relatively abundant in biological processes such as apoptotic processes and cytokine-mediated signaling pathways (Figure S2). Overall, the results of this study demonstrate the heterogeneous distribution of T-cell subpopulations in the mouse lacrimal glands.

### 2.3. ILCs in Mouse ELGs

**Figure 3:**
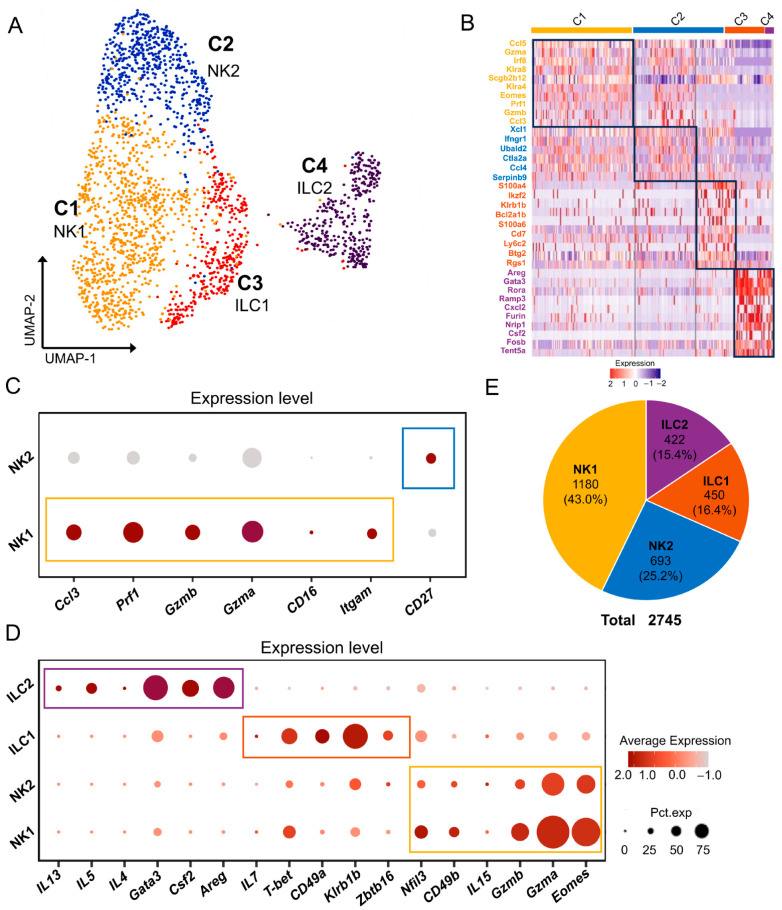
ILCs in mouse lacrimal glands. (**A**) UMAP visualization of ILC subpopulations in mouse ELGs. (**B**) Heatmap of Z scores for scaled expression values of DEGs for each cluster. Colors are based on the natural logarithm of normalized RNA expression. The black boxes indicate the DEGs within each cluster. (**C**) Dot plot showing marker genes in NK1s and NK2s cells. The dot size denotes the percentage of cells expressing the marker gene in the respective cell population, and the dot color denotes the natural logarithm of normalized RNA expression. Different colored boxes represent different cell clusters. (**D**) Dot plot showing marker genes in each cluster of cells. The dot size represents the percentage of cells expressing the marker gene in that cell population, and the dot color is based on the natural logarithm of normalized RNA expression. Different colored boxes represent different cell clusters. (**E**) Pie chart showing the number of cells in each subpopulation of ILCs and their respective percentage of the ILC population.

To understand the characteristics of ILC subpopulations in the mouse ELGs, we further divided the 2745 ILCs screened in the four samples into subpopulations by a second round of combined clustering analysis. UMAP dimensionality reduction analysis yielded four clusters of ILCs (Figure 3A,B). Among these four clusters, Cluster 1 and Cluster 2 were annotated as NKs due to the expression of the NK-specific transcription factor *Eomes* [62]. In agreement with the literature, Cluster 1 was annotated as NK1s due to the relatively high expression of *Itgae* (encoding CD11b) and low expression of *CD27*, while Cluster 2 was annotated as NK2s due to the low expression of *Itgae* and high expression of *CD27*. Functional enrichment analysis using KEGG revealed that the genes expressed in the NK1 cell cluster were primarily enriched in signaling pathways related to natural killer cell cytotoxicity, apoptosis, and necroptosis. GO-BP analysis further demonstrated enrichment in biological processes associated with NK cell function, such as cell activation involved in immune response, response to cytokine, and positive regulation of gene expression (Figure S3). Functional KEGG analysis of the NK2 cell cluster revealed that the genes expressed predominantly in this cluster were enriched in pathways related to natural killer cell-mediated cytotoxicity and interactions with cytokines (viral protein interaction with cytokine and cytokine receptor, cytokine-cytokine receptor interaction). GO-BP analysis showed that the genes expressed in the NK2 cluster were primarily enriched in biological processes such as viral gene expression and establishment of protein localization at the membrane (Figure S3).

Cluster 3 highly expresses the ILC1 marker genes *Klrb1c* (encoding CD161, Nk1.1) and *Ncr1* (encoding Nkp46) [63] and was therefore annotated as an ILC1 subset (Figure 3C,D). Functional analysis using KEGG revealed that the genes expressed in the ILC1 cell cluster were primarily enriched in signaling pathways related to apoptosis, NF-kappa B, and TNF. GO-BP analysis further demonstrated that genes expressed in the ILC1 cell cluster are involved in biological processes related to immune cell adhesion and activation (Figure S3). 

Cluster 4 expresses the classical ILC2 markers *Areg*, *Csf2*, and *Gata3* [23,64] and therefore belongs to the ILC2 subgroup (Figure 3C,D). Functional enrichment analysis using KEGG revealed that the genes expressed in the ILC2 cell cluster were primarily enriched in TNF- and IL-17-associated signaling pathways. GO-BP analysis further demonstrated enrichment in biological processes such as the apoptotic process, positive regulation of the nucleobase-containing compound metabolic process, and positive regulation of the biosynthetic process (Figure S3). Figure 3E shows the total number of cell clusters of these four groups and their respective percentages of all ILC cell populations in mouse ELGs. In conclusion, our research findings demonstrate the heterogeneous distribution of ILCs in mouse lacrimal glands, suggesting their potential involvement in various immunological physiological processes.

### 2.4. MΦs in Mouse ELGs

**Figure 4:**
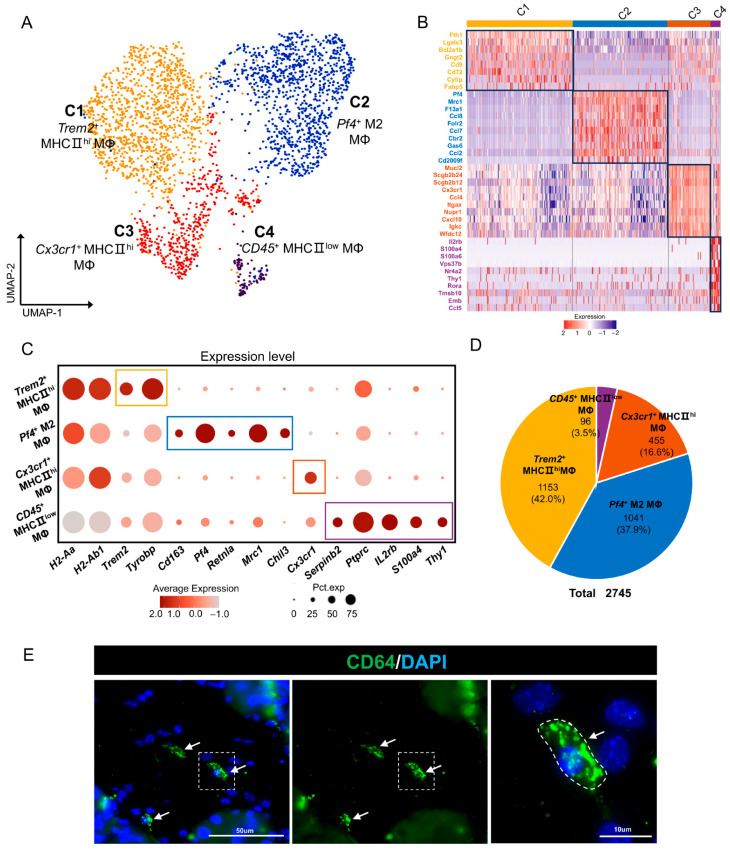
MΦs in mouse ELGs. (**A**) UMAP visualization of MΦ subpopulations in mouse ELGs. (**B**) Heatmap of Z scores for scaled expression values of DEGs for each cluster. Colors are based on the natural logarithm of normalized RNA expression. The black boxes indicate the DEGs within each cluster. (**C**) Dot plot showing the marker gene in each cluster. The dot size indicates the percentage of cells expressing the marker gene in the respective cell population, and the dot color indicates the natural logarithm of the normalized RNA expression. Different colored boxes represent different cell clusters. (**D**) Pie chart showing the number of cells in each subpopulation of MΦs and their respective percentage of the total MΦ population. (**E**) Histological immunofluorescence staining of the lacrimal gland showing FITC-conjugated anti-mouse CD64^+^ (green) antibody-positive cells. White arrows point to positively stained cells, scale bar = 50 µm and high magnification image scale is 10 µm.

To characterize the MΦ subpopulations in the mouse ELGs, we first utilized UMAP downconversion to analyze the 2745 MΦs obtained from the four samples. Through a second round of cluster analysis, our dataset identified four distinct clusters (Figure 4A,B). Cluster 1, referred to as *Trem2*^+^ MHC II^hi^ MΦ, was characterized by its high expression of MHC class II antigens, specifically *H2-Aa* and *H2-Ab1*. Additionally, this cluster exhibited elevated expression of *Tyrobp* (encoding DAP12), *Trem2* (encoding TREM-2), and *Fth1* (Figure 4C). These molecular signatures were indicative of the *Trem2*^+^ MHC-II^hi^ MΦ subpopulation [31,65,66]. Functional KEGG enrichment analysis revealed that the genes expressed in the *Trem2*^+^ MHC II^hi^ MΦ cluster were mainly enriched in cell engulfment and death-related signaling pathways, such as the lysosome, phagosome, ferroptosis, and pentose phosphate pathways. Additionally, GO-BP analysis showed that these cells were predominantly enriched in the cellular amide metabolic process, cellular macromolecule catabolic process, and peptide metabolic process, indicating their involvement in cellular molecular degradation and metabolic processes (Figure S4).

Cluster 2 demonstrated pronounced expression of *44* as well as high expression of the M2 MΦ marker genes *Mrc1* (encoding mannose receptor 1, CD206), *Cd163* (encoding macrophage scavenger receptor CD163), *Retnla* (encoding resistin-like molecule alpha (RELMα)), and *Chil3* (*Chi3l3*, *Ym1*) [67,68,69,70], leading to its classification as a *Pf4*^+^ M2 MΦ (Figure 4C). Functional KEGG enrichment analysis revealed that the genes expressed in this subpopulation of cells were mainly enriched in cell-engulfment-related signaling pathways, such as endocytosis and phagosomes, as well as the MAPK signaling pathway. Additionally, GO-BP analysis showed that these cells were enriched in immune effector processes, such as the defense response, regulation of immune system processes, and response to oxygen-containing compounds (Figure S4). 

Cluster 3, characterized by its high expression of *Cx3cr1* and MHC-II class II antigens, was designated *Cx3cr1*^+^MHCII^hi^ MΦ [71] (Figure 4C). Functional KEGG enrichment analysis revealed that the genes expressed in the *Cx3cr1*^+^MHCII^hi^ MΦ cluster were mainly enriched in Toll-like receptor and MAPK signaling pathways. Additionally, GO-BP analysis showed that these cells were enriched in apoptotic signaling pathways, regulation of cell death, and response to abiotic stimulus biological processes (Figure S4). 

Finally, Cluster 4 was identified as the *CD45*^+^ MHCII^low^ MΦ subpopulation [25,72]. This classification was based on its elevated expression levels of *Serpinb2* and *Ptprc*, along with lower levels of MHC-II class antigen (Figure 4C). Functional KEGG enrichment analysis revealed that the genes expressed in this subpopulation of cells were predominantly enriched in signaling pathways related to spliceosomes and NF-κB. Conversely, GO-BP analysis demonstrated significant enrichment in cellular processes involved in the metabolic breakdown of biological substances, such as the mRNA metabolic process, macromolecule catabolic process, and organic cyclic compound catabolic process (Figure S4). Figure 4D provides an overview of the total number and respective percentages of these four MΦ clusters within the overall MΦ population. Furthermore, we validated the presence of MΦs using immunofluorescence and the MΦ-specific marker CD64 (Figure 4E) [73]. In conclusion, our findings have revealed the presence of heterogeneous subpopulations of MΦs in the mouse ELG, which are probably involved in various immunological processes.

### 2.5. DCs/pDCs in Mouse ELGs

**Figure 5:**
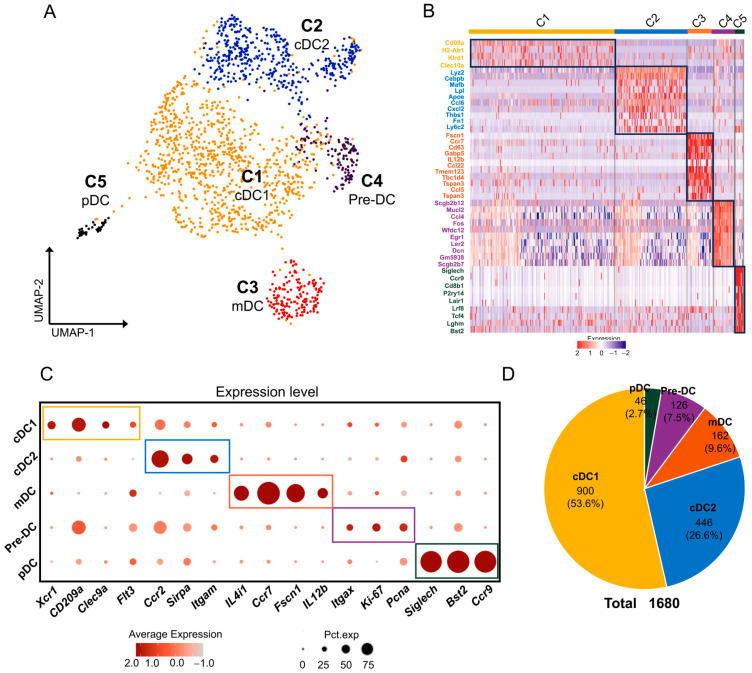
DC/pDCs cells in mouse ELGs. (**A**) UMAP visualization of a subcluster of 1680 DCs in mouse ELGs. (**B**) Heatmap of Z scores for scaled expression values of DEGs for each cluster. Colors are based on the natural logarithm of normalized RNA expression. The black boxes indicate the DEGs within each cluster. (**C**) Dot plot showing the signature marker genes in each cluster of cells. The dot size denotes the percentage of cells expressing the marker gene in the respective cell population, and the dot color denotes the natural logarithm of normalized RNA expression. Different colored boxes represent different cell clusters. (**D**) Pie charts showing the number of cells in each subpopulation of DC/pDCs and their respective percentages in the total DC populations.

To gain a better understanding of the characteristics of DC/pDC subpopulations in mouse exocrine lacrimal glands (ELGs), we conducted a comprehensive analysis by examining 1680 DC/pDC cells from four samples using a second round of clustering analysis. Through UMAP downscaling analysis, we were able to identify five distinct clusters (Figure 5A,B). Cluster 1 was classified as conventional type 1 dendritic cells (cDC1) based on the expression of *Xcr1* (encoding chemokine receptor 1), *CD209a*, *Clec9a* (encoding C-type lectin domain family 9 member A), and *Flt3* (encoding Fms-related tyrosine kinase 3), which are well-established markers of cDC1 [74,75] (Figure 5C). KEGG analysis of the expressed genes in this cluster showed significant enrichment in antigen processing and presentation, apelin, and prolactin signaling pathways, while GO-BP analysis revealed a predominant involvement of cDC1 cells in biosynthetic and metabolic processes such as the cellular amide metabolic process, peptide metabolic process, and mRNA metabolic process (Figure S5).

Cluster 2 was identified as conventional type 2 dendritic cells (cDC2) due to the high expression levels of *Sirpα*, *Ccr2*, and *Itgam* [76] (Figure 5C). KEGG analysis of the expressed genes in this cluster showed significant enrichment in cell-engulfment-related signaling pathways such as lysosome, phagosome, and F gamma R-mediated phagocytosis, while GO-BP analysis revealed predominant enrichment in inflammatory and immune response processes such as the immune effector process, cell activation involved in the immune response, myeloid-leukocyte-mediated immunity, the innate immune response, and the inflammatory response (Figure S5).

Cluster 3 exhibited characteristics of migratory dendritic cells (mDCs), as it specifically overexpressed *Ccr7*, *Il4i1*, *IL12b*, and *Fscn1* [75] (Figure 5C). KEGG analysis of the expressed genes in this cluster showed significant enrichment in C-type lectin receptor, chemokine, and NF-kappa B signaling pathways, while GO-BP analysis revealed predominant enrichment in immune effector processes and antigen processing as well as presentation biological processes such as the immune effector process, regulation of the immune response, antigen processing, and presentation of exogenous peptide antigen via MHCII (Figure S5).

In contrast, Cluster 4 was designated a pre-DC subpopulation due to the high expression of the proliferative genes *Mki67* (encoding Ki-67), *Pcna*, and *Itgax* [77,78] (Figure 5C). KEGG analysis of the expressed genes in this cluster showed significant enrichment in antigen processing and presentation, MAPK, and TNF signaling pathways, while GO-BP analysis revealed a predominant involvement in biological processes such as the response to cytokine, regulation of the cellular response to stress, and the apoptotic process (Figure S5).

Finally, Cluster 5 exhibited characteristics of plasmacytoid dendritic cells (pDCs), as it specifically expressed the genes *Siglech*, *Bst2*, and *Ccr9* [35,79,80,81] (Figure 5C). KEGG analysis of the expressed genes in this cluster showed significant enrichment in the Toll-like receptor signaling pathway, while GO-BP analysis revealed predominant enrichment in immune response processes such as the innate immune response, regulation of the immune response, and the defense response to viruses (Figure S5). Figure 5D demonstrates the total count and corresponding proportions of these five cell clusters among the DC population. Taken together, our results suggest the presence of a diverse subset of DCs within the mouse lacrimal gland. These distinct cell subpopulations may play roles in various immune regulatory processes and immunological biological events within the lacrimal gland.

### 2.6. MC/Basophils in Mouse ELGs

**Figure 6:**
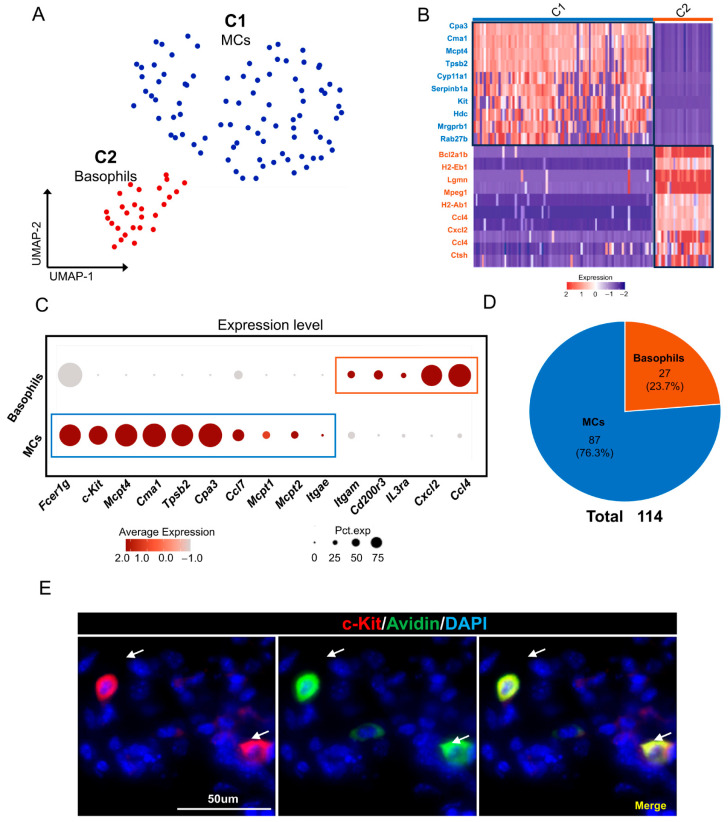
MC/basophils in mouse ELGs. (**A**) UMAP visualization of 114 cells of the MC/basophil subpopulation in mouse ELGs. (**B**) Heatmap of Z scores for scaled expression values of DEGs for each cluster. Colors are based on the natural logarithm of normalized RNA expression. The black boxes indicate the DEGs within each cluster. (**C**) Dot plot showing the signature marker gene in each cluster of cells. The dot size indicates the percentage of cells expressing the marker gene in the respective cell population, and the dot color indicates the natural logarithm of the normalized RNA expression. Different colored boxes represent different cell clusters. (**D**) Pie chart showing the number of cells in each subpopulation of MC/basophils and their respective percentages in the total MC/basophil cell population. (**E**) Histological immunofluorescence staining of the lacrimal gland showing mast cells stained with FITC-conjugated avidin (green) and PE-conjugated anti-c-Kit (red). White arrows point to positively stained cells, scale bar = 50 µm.

We conducted a comprehensive analysis to elucidate the characteristics of the MC/basophil subpopulation in the mouse ELGs. A total of 114 MC/basophils were screened from four samples, and a second round of cluster analysis was performed. This analysis resulted in the generation of two distinct clusters through UMAP dimensionality reduction analysis (Figure 6A,B). Cluster 1 cells were found to express *FcεRI* and *c-Kit* (*CD177*), indicating their mast cell identity. These cells also exhibited high expression levels of classical mast cell proteases, such as *Mcpt4*, *Mcpt5*, *Mcpt6*, variant carboxypeptidase A3 (*Cpa3*), *Mcpt1*, *Mcpt2*, and *Itgae* (Figure 6C) [82]. Additionally, Cluster 1 cells displayed significant expression of the chemokine ligand *Ccl7* [83,84] (Figure 6C). Thus, these cells were designated mast cells (MCs). The KEGG functional enrichment analysis of the MC-expressed genes revealed significant enrichment in signaling pathways related to dopaminergic synapses and serotonergic synapses, as well as hormone-related pathways such as parathyroid hormone synthesis, secretion, and action and the estrogen signaling pathway. Additionally, analysis of the gene ontology biological process indicated significant enrichment in biological processes associated with various stimulus responses, including response to endogenous stimulus, response to oxygen-containing compound, and response to hormone (Figure S6).

Cluster 2 cells shared several coexpressed genes with mast cells, but they exhibited stronger expression of *Il3rα* (*CD123*) and *c-Kit* (*CD177*) than mast cells [85]. Moreover, Cluster 2 cells displayed high expression levels of the chemokines *Ccl4* and *Cxcl2* [86,87] (Figure 6C). Therefore, Cluster 2 was identified as a basophil subpopulation. KEGG functional enrichment analysis of genes expressed in this cell population revealed significant enrichment in pathways associated with cell engulfment processes such as phagosome, lysosome, and apoptosis. Conversely, GO-BP analysis revealed enrichment in biological processes associated with the immune response, defense and response to bacterial stimuli, such as the defense response, immune effector process, cell activation involved in the immune response, and the cellular response to molecules of bacterial origin (Figure S6). To validate the presence of mast cells in the lacrimal gland tissue, we further performed immunofluorescence staining using an anti-c-Kit antibody in conjunction with avidin staining (Figure 6E). This analysis further confirmed the presence of c-Kit-positive mast cells throughout the lacrimal gland tissue. In conclusion, our findings indicate the existence of a distinct subpopulation of MCs/basophils in the mouse ELG.

### 2.7. B Cells/Plasma Cells in Mouse ELGs

**Figure 7:**
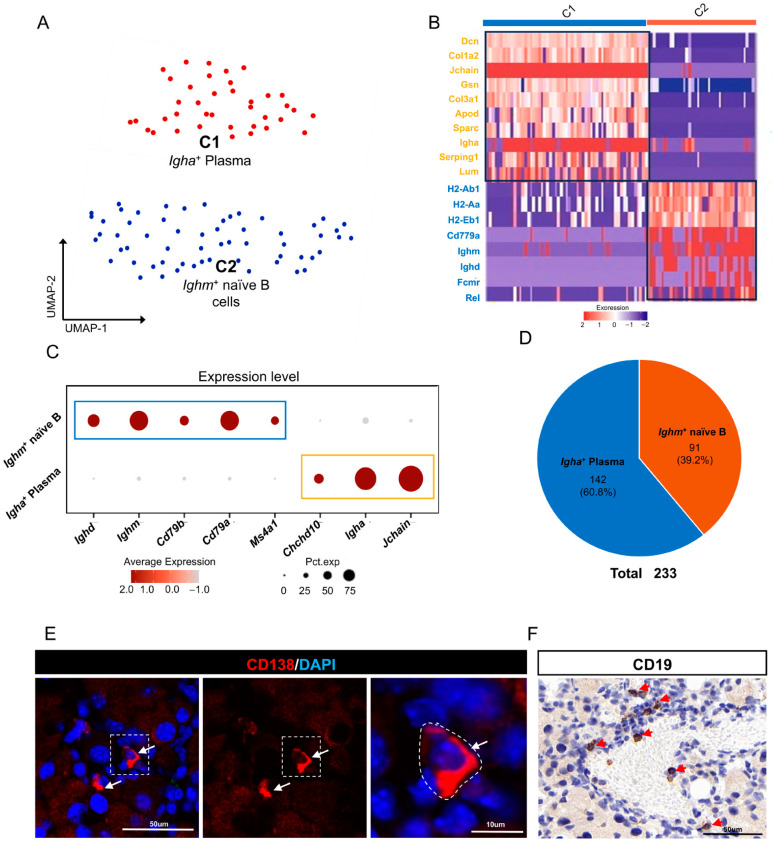
B cells/plasma cells in mouse ELGs. (**A**) UMAP visualization of a subcluster of 233 B/plasma cells in mouse ELGs. (**B**) Heatmap of Z scores for scaled expression values of DEGs for each cluster. Colors are based on the natural logarithm of normalized RNA expression. The black boxes indicate the DEGs within each cluster. (**C**) Dot plot showing the signature marker gene in each cluster of cells. The dot size denotes the percentage of cells expressing the marker gene in the respective cell population, and the dot color denotes the natural logarithm of normalized RNA expression. Different colored boxes represent different cell clusters. (**D**) Pie charts showing the respective percentages of B cells and plasma cells. (**E**) Histological immunofluorescence staining of the lacrimal gland showing plasma cells stained with PE-conjugated anti-CD138 (red), white arrows point to positively stained cells, scale bar = 50 µm and high magnification image scale is 10 µm. (**F**) Histological immunofluorescence staining for the lacrimal gland, showing B cells using an anti-mouse CD19 antibody, red arrows point to positively stained cells, scale bar = 50 µm.

To elucidate the characteristics of the B-cell/plasma cell subpopulation in the mouse ELG, we screened 233 B cells/plasma cells from four samples using a second round of clustering analysis. This analysis, which involved UMAP nonlinear dimensionality reductions, resulted in the identification of two distinct cell clusters (Figure 7A,B). Cluster 1, which expressed high levels of *Jchain* and *Igha* (encoding IgA), was labeled *Igha*^+^ plasma cells [88,89]. On the other hand, Cluster 2 cells exhibited high levels of *Ms4a1*, *Cd79a*, *Cd79b*, *Ighm*, and *Ighd* but low levels of *Chchd10* [90,91,92,93]. These cells were therefore labeled *Ighm*^+^ naive B cells (Figure 7C). Figure 7D illustrates the total number and percentages of these two cell subsets within the B-cell/plasma cell population in the mouse ELGs. Further analysis using GO-BP revealed that genes expressed by *Igha*^+^ plasma cells were primarily associated with an inflammatory response, response to endogenous stimulus, response to oxygen-containing compound, blood vessel morphogenesis, and other processes (Figure S7). In contrast, IgM^+^ naive B cells were enriched for nuclear-transcribed mRNA catabolic processes, B-cell receptor signaling pathways, cotranslational protein targeting to membrane defense response to bacteria, and other biological processes (Figure S7). Collectively, our findings suggest the presence of a distinct B-cell/plasma subpopulation in mouse ELGs.

### 2.8. Analysis of Cell-Cell Communication between Immune Cell Populations in Mouse Lacrimal Glands

**Figure 8:**
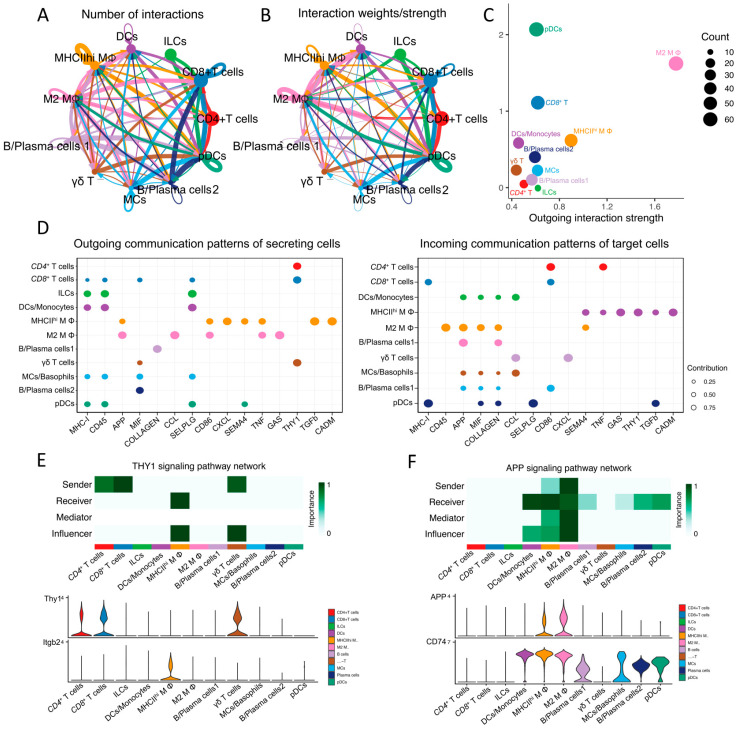
CellChat-inferred cell–cell communication networks reveal the functional heterogeneity of ELG immune cell populations. (**A**) Circle network plots displaying the number of interactions between cells, generated using CellChat. The thickness of the lines is proportional to the number of interactions between cells. The size of the nodes is proportional to the number of different cell types. (**B**) Circle network plots display the weights/strength of interactions between cells, generated using CellChat. The thickness of the lines is proportional to the weights/strength of interactions between cells. (**C**) Visualization of the primary senders and receivers in a 2D spatial context generated by CellChat demonstrates the interactions between cells. (**D**) Dot plots display the most important outgoing (left) and incoming (right) signaling patterns in ELG immune cell clusters. The size of the dots is proportional to the contribution score obtained from pattern recognition analysis. A higher contribution score indicates a richer signaling pathway in the corresponding cell clusters. (**E**) Heatmaps display the inferred communication network of the Thy1 signaling pathway among all cell types, where darker colors represent stronger involvement of the corresponding signaling pathway in the cell clusters. Violin plots show the distribution of signal gene expression in each signaling network. (**F**) Heatmaps display the inferred communication network of the APP signaling pathway among all cell types, where darker colors represent stronger involvement of the corresponding signaling pathway in the cell clusters. Violin plots show the distribution of signal gene expression in each signaling network.

To elucidate the qualitative and quantitative aspects of potential interactions among immune cells in mouse lacrimal glands (ELGs), we initially uploaded the Seurat object to construct and quantify the global signaling cross-talk atlases using CellChat [94]. As depicted in Figure 8A,B, a complex and intricate network of intercellular interactions exists among immune cell subpopulations within mouse ELGs. Furthermore, we employed scatter plots to visualize the primary cell signaling senders and receivers within a 2D spatial context. The results revealed that the interactions between pDCs, M2 MΦs, and *CD8*^+^ T cells were the most prominent (Figure 8C).

To investigate the signaling pathways involved in intercellular communication among ELG immune cell populations, pattern recognition analysis was employed. This analysis aimed to study the most significant outgoing and incoming signaling pathways in these populations, as shown in Figure 8D. In particular, we focused on one major signaling pathway with high communication strength for T cells (*CD4*^+^ T cells, *CD8*^+^ T cells, and γδ T cells) and MΦ populations, as depicted in Figure 8E,F.

The heatmap of the inferred thymus cell antigen 1 (Thy1, CD90) signaling network revealed interesting findings. This result indicated that T cells were the most prominent sources of Thy1 signaling pathway ligands, while MΦs were the major signaling targets of the Thy1 signaling pathway. This information is visually represented in Figure 8E. Furthermore, the expression of Thy1 signaling genes was predominantly observed in T-cell subgroups, as shown in the violin plots (Figure 8E).

Moving on to another signaling pathway, the heatmap of the inferred amyloid precursor protein (APP) signaling network highlighted MΦs as the most prominent sources of this pathway. Interestingly, MΦs were also major targets of the APP signaling pathway. Additionally, the APP signaling targets included DCs, pDCs, and B cells/plasma cells, as depicted in Figure 8F.

## 3. Discussion

**Figure 9:**
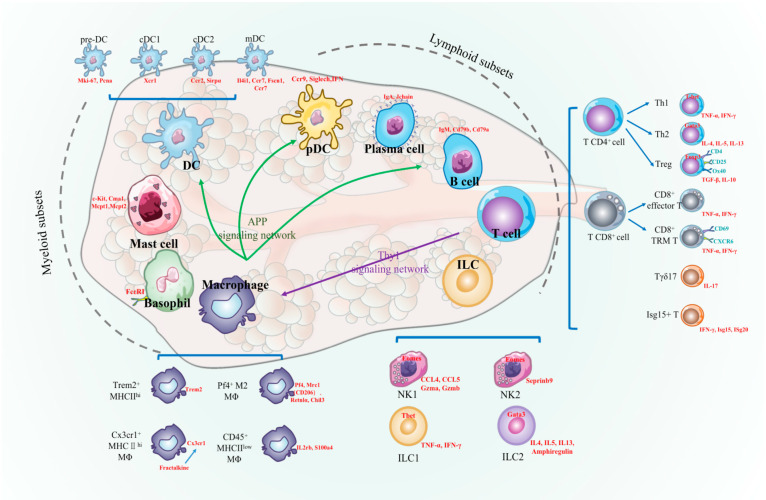
Immune cell diversity in the lacrimal glands of healthy mice.

By employing single-cell RNA sequencing, we have effectively ascertained and characterized the distinct subsets of immune cells that exist in the lacrimal glands (ELGs) of mice under normal conditions. Our observations unveil a significant and heterogeneous assemblage of immune cells within the lacrimal gland (Figure 9). These findings not only provide unprecedented insights into the immune microenvironment of the lacrimal gland but also present potential avenues for comprehending the underlying mechanisms of lacrimal gland-related disorders and devising novel preventive and therapeutic strategies in the future.

### 3.1. T Cells in Mouse Lacrimal Glands

Consistent with previous findings [7,95], our research confirms a variety of T-cell subsets within the mouse lacrimal gland at a single-cell resolution. We discovered mitotic *CD4*^+^ T cells, which probably serve as precursors to diverse *CD4*^+^ lineages critical for local immune modulation. These precursor cells are poised to differentiate into distinct subsets—Th1, Th2, Th17, and Treg—each with a specialized role: Th1 cells drive cellular immunity, Th2 cells propel antibody production, Th17 cells mediate inflammatory processes, and Treg cells maintain immune balance by preventing overactive responses [96].

Our study discovered two distinct populations of *CD8*^+^ T cells in the lacrimal glands of mice: *CD8*^+^ cytotoxic T cells and *CD8*^+^ tissue-resident memory T cells. The significance of the former is evident in their ability to control viral infections and eliminate tumors [97]. On the other hand, the latter represents a specific subset of memory T cells that reside in nonlymphoid tissues and provide rapid and long-lasting protection against reinfection via rapid immune response, cytotoxicity, cytokine production, and tissue repair [98]. This division of *CD8*^+^ T-cell functionality highlights the complexity and sophistication of the immune landscape within the lacrimal gland, revealing potential pathways for antiviral and immunoregulatory mechanisms.

Our study also identified the presence of *CD4*^+^ and *CD8*^+^ *Isg15*^+^ T-cell clusters in the mouse lacrimal gland, in addition to the classical cell subsets. The classification of *Isg15*^+^ T-cell clusters as a specific subset is not universally recognized, however. Typically in the research, the observation of *Isg15* expression in T cells aims to explore how T cells respond to IFN signaling in the context of inflammation and tumors [56,99]. Despite this, the physiological and pathological significance of this particular population appearing in the lacrimal gland remains unclear.

Unlike αβT cells that acquire peripheral effector functions, γδ T cells undergo primary development within the thymus, attaining maturity and differentiating into effector cells. Within the thymus, the recombination of different types of Vγ (Vγ2,3,4,5,8,9) and Vδ chains of TCRs in γδ T cells gives rise to subsets with distinct functionalities and characteristics [100]. Subsequently, upon thymic maturation, these subsets of γδ T cells exit the thymus and settle in various tissues and organs at different developmental stages [101]. Thus, γδ T cells can rapidly respond to pathogen infections, inflammation, and tissue damage [102]. To date, the γδ T cells we recognize mainly fall into two categories: Tγδ1 cells that produce IFN-γ and Tγδ17 cells that produce IL-17 [103]. These cells express the key transcription factors T-bet and RORγt [102]. In our study, we identified a predominant population of Tγδ17 cells in the lacrimal glands, chiefly belonging to the Vγ4 subset. This corresponds with the γδ T cells distributed in the lungs, dermis, and lymph nodes, which share the same origins and features [102,104]. Previous research has indicated that the Vγ4 subset exhibits immunoregulatory inhibitory effects [105]. However, the exact physiological roles of these cells in the lacrimal glands remain elusive.

### 3.2. ILCs in Mouse Lacrimal Glands

ILCs constitute a family of lymphocytes originating from common lymphoid progenitors in the bone marrow [106]. They are considered the innate counterpart of T cells, as they lack antigen-specific receptors such as T cells but can rapidly respond to pathogens and tissue damage. ILCs are widely distributed throughout various tissues and organs, including the cornea and conjunctiva [25,107]. ILCs comprise five different subsets: cytotoxic natural killer cells (NK cells), lymphoid tissue inducer cells, and three helper-like subsets, ILC1s, ILC2s, and ILC3s [108,109]. Notably, we identified NKs, ILC1s, and ILC2s in the mouse lacrimal gland. These lacrimal gland NK cells could be further subdivided into two distinct groups, NK1s and NK2s, exhibiting similarities to findings in both human and mouse spleens and blood [62]. Among these, NK1 cells displayed elevated expression levels of key genes, including chemokines (*Ccl4* and *Ccl5*), the IFN regulatory factor *Irf8*, and NK activation receptors (*Klrb1c*, *Klra8*, and *Klra4*). Additionally, NK1s exhibited higher levels of *Gzma* and *Gzmb*, indicating increased cytotoxicity and a more active phenotype compared with NK2 cells. Interestingly, our data also unveiled a subset of NK2 cells characterized by high expression of *Serpinb 9*, an inhibitor of endogenous granzyme B. This particular protein plays a crucial role in safeguarding NK/T cells from granzyme B-induced cell death [110].

ILC1s, which are a specific type of innate lymphoid cell, play a vital role in protecting the host against intracellular pathogens [111]. When stimulated by specific cytokines, such as IL-12 and IL-18, ILC1s produce IFN-γ. These cells are primarily located at epithelial barrier sites and exhibit a rapid response to infection, often preceding the activation of cytotoxic NKs [112]. This suggests that ILC1s in the lacrimal gland contribute to the defense against intracellular pathogens, highlighting their significance in the initial stages of host defense.

In contrast, ILC2s are known for their ability to produce certain cytokines, including IL-4, IL-5, and IL-13, in response to various cytokines, namely, thymic stromal lymphopoietin, IL-25, and IL-33 [113]. These cytokines secreted by ILC2s play a critical role in promoting mucosal and barrier immunity, providing protection against parasites and acting as triggers for allergic reactions [114]. ILC2s also produce amphiregulin, a member of the epidermal growth factor family. Amphiregulin stimulates the proliferation and differentiation of epithelial cells, thereby facilitating tissue repair after injury [115]. It is therefore plausible that ILC2s present in the lacrimal gland may perform similar functions. However, further research is needed to clarify this hypothesis.

### 3.3. MΦs in Mouse Lacrimal Glands

MΦs play a critical role in various biological processes, including immune responses, inflammation, tissue repair, and immune regulation [116]. In this study, we identified four distinct subpopulations of MΦs within the lacrimal glands of mice. Among these subpopulations, the Trem2^+^MHCII^hi^ MΦ group stands out due to its high Trem2 expression. Research indicates that Trem2, expressed in microglia, plays a key role in clearing neuronal debris and exhibits anti-inflammatory properties [117,118]. Additionally, Trem2^+^ MΦs have been found to enhance NK cell activity and suppress tumor cell growth by modulating interleukin interactions and production in pathological conditions [66,119]. Notably, the loss of Trem2 function in MΦs leads to increased IFN-γ-induced immune activation, a proinflammatory shift, and enhanced tumor-cell-killing capacity [66].

Another notable group of MΦs is the *Pf4*^+^ M2 MΦ subset, which can be found in various normal organs and tissues [120,121,122]. This subset is classified as M2 MΦs due to its high expression of key M2 marker genes, such as *Mrc1* (*CD206*), *Retnlα* (*Fizz1)*, and *Chil3* (*Chi3l3*, *Ym1*) [67,68]. M2 MΦs play a crucial role in maintaining homeostasis, tissue remodeling, and metabolic adaptation [123].

The *Cx3cr1*^+^MHCII^hi^ MΦ subpopulation, characterized by its high *Cx3cr1* expression as a receptor for the chemokine fractalkine, plays a critical role in MΦ function at sites of inflammation and tissue damage [124,125]. Fractalkine, a chemokine expressed on endothelial cells, is involved in the recruitment and retention of *Cx3cr1*^+^ MΦs at inflammatory sites. These MΦs are known to have a proinflammatory phenotype and are involved in the clearance of apoptotic cells and debris [126,127]. Additionally, the *Cx3cr1*^+^ subset has been shown to promote tissue repair and regeneration through the production of growth factors and cytokines [71,128].

Furthermore, we identified a *CD45*^+^ MHCII^low^ MΦ subset in the lacrimal gland. This subset is characterized by high expression of *IL2rb* (encoding CD122), S100 calcium binding protein A4 (*S100a4*), and *Thy1*. IL2rb, the receptor for interleukin-2 (IL2), is expressed in a high-affinity form on activated MΦs and is involved in regulating autoimmune responses and normal lymphocyte development [129,130]. Additionally, *S100a4* influences the chemotaxis of MΦs under inflammatory conditions and their infiltration toward inflammatory sites [131]. Furthermore, Thy1 plays a crucial role not only in MΦ-driven inflammatory responses but also in other physiological processes [132].

Overall, the identification and characterization of these distinct subpopulations of MΦs provide valuable insights into their diverse functions and potential therapeutic targets for various pathological conditions. Despite these findings, the specific physiological roles of these MΦ populations in the lacrimal gland remain unclear.

### 3.4. DC/pDCs in Mouse Lacrimal Glands

DCs, a diverse group of immune cells, play a critical role in antigen presentation and immune response regulation. These cells are present in various tissues, including lacrimal glands [11,133]. In our studies of mouse lacrimal glands, we first discovered the presence of predendritic cells (pre-DCs), which are the precursors of DCs. These cells exist in the tissue and develop into mature dendritic cells, specifically cDC1 and cDC2, during the maturation process [134]. These cell populations are characterized by the expression of proliferation-related genes, particularly *mki67*. Additionally, we predictively identified two major subpopulations of cDCs, cDC1 and cDC2, based on their marker gene expression profiles. cDC1s excel in cross-presentation, a process in which they capture exogenous antigens and present them on MHC class I molecules to activate *CD8*^+^ T cells [135,136]. They are particularly effective in initiating cytotoxic T-cell responses against viral and tumor antigens. On the other hand, cDC2s participate in antigen presentation to *CD4*^+^ T cells, playing a pivotal role in promoting immune responses mediated by Th1, Th2, and Th17 cells under specific circumstances [137,138,139,140].

mDCs have the unique ability to migrate from peripheral tissues to draining lymph nodes, carry tissue autoantigens, and play distinct roles in immune responses, particularly in inflammatory conditions. In our observations of mDCs in mouse lacrimal glands, we noted high expression of *Il4i1*, *Ccr7*, and *Fscn1* in these cell populations [25]. *Il4i1*, a signature gene of IL-4, is considered a marker of regulatory DCs and has the capacity to inhibit the appearance of IFN-γ-secreting T cells. Moreover, migratory DCs express CC-chemokine receptor 7 (CCR7), a crucial molecule involved in their migration to draining lymph nodes [141]. This suggests that migratory DCs in the lacrimal glands may have a regulatory role, as indicated by the expression of *Il4i1* and *Ccr7*. Additionally, the presence of Fascin-1 (Fscn1), an actin-bundling protein, in migratory DCs suggests their potential involvement in antigen presentation and T-cell activation [142]. Therefore, based on the expression of these markers, it is likely that the migratory DCs in mouse lacrimal glands are regulatory in nature and contribute to immune regulation and tolerance.

In contrast, pDCs represent a rare and specialized subgroup of DCs primarily located in peripheral blood and lymphoid tissues [143]. These distinctive cells play a pivotal role in the immune response against viral infections by producing substantial quantities of type I IFNs [79]. Moreover, pDCs actively participate in interactions with other immune cells, aiding in the initiation of adaptive immune responses. They also contribute to immune regulation and tolerance mechanisms [144]. Interestingly, our data revealed the presence of a small pDC population in the lacrimal gland, characterized by the expression of classical markers such as *Siglech* and *Ccr9*. However, the physiological significance of these pDCs within the lacrimal gland remains unexplored. Therefore, further investigation is warranted to elucidate the functional role of pDCs within the lacrimal gland and their potential impact on ocular immunity.

### 3.5. Mast Cells and Eosinophils in Mouse Lacrimal Glands

MCs have their origins in hematopoietic progenitor cells situated within the bone marrow; they subsequently traverse the bloodstream to infiltrate various tissues, where they undergo differentiation and acquire distinctive granular MC phenotypes, a process heavily influenced by the local microenvironment. MCs are categorized among tissue-resident immune cells, primarily localized in proximity to blood vessels, nerves, secretory glands, connective tissues, and mucosal barriers. Notably, MCs have also been observed in tissues such as the lacrimal gland in both humans and mammals, where they play essential roles in the normal development and maintenance of these tissues [145,146] and contribute to age-related responses and disease processes [145,146,147]. In this study, a distinct population of MCs with prominent features was identified within the lacrimal glands of mice at single-cell resolution.

In contrast, basophils, while also originating from the bone marrow, undergo maturation within the confines of the bone marrow itself before their release into the peripheral circulation. Basophils express high-affinity IgE receptors (FcεRI) on their cell surfaces and exhibit rapid release of granule contents upon receptor cross-linking. While the presence of basophils within the lacrimal gland was established in the present study, their exact roles in maintaining lacrimal gland homeostasis and their involvement in certain disease processes require further rigorous investigation and elucidation.

### 3.6. B Cells/Plasma Cells in Mouse Lacrimal Glands

B cells and plasma cells residing within secretory glands, including the lacrimal glands, play pivotal roles in orchestrating local immune responses and antibody production [8]. This study delineates two distinct subsets within the lacrimal gland: IgM^+^ naive B cells and IgA^+^ plasma cells. IgM^+^ naive B cells constitute a distinct subset within the spectrum of B lymphocytes. These cells originate from B-cell precursors in the bone marrow and undergo a series of differentiation and maturation stages before their establishment. Upon encountering extraneous antigens, IgM^+^ naive B cells can be activated through the engagement of their B-cell receptors with these antigens. Once activated, they commence a process of proliferation and differentiation, eventually transforming into plasma cells responsible for antibody production. Moreover, B cells regulate the magnitude and direction of immune responses by secreting cytokines and antibodies into exocrine glands. In contrast, plasma cells act as effector cells within the lacrimal gland niche, secreting antibodies directly into the tear fluid. This secretion facilitates pathogen neutralization, antigen clearance, and the promotion of inflammatory responses, among other essential functions [148]. However, it must be emphasized that further research is needed to fully understand the differentiation processes of these two distinct B-cell subpopulations in the lacrimal gland and clarify their specific functional repertoires.

### 3.7. Cell–Cell Signaling among Immune Cells

CellChat provides a state-of-the-art method for inferring intercellular signaling networks from scRNA-seq data [94]. Similar to other ocular tissues [25,149], there is sophisticated crosstalk between immune cells in the context of ELGs. In particular, M2-MΦ, *CD8*^+^ T cells, and pDCs emerge as the key cell groups involved in both incoming and outgoing signals. Looking more closely, the Thy1 signaling network, particularly within lacrimal gland T cells, shows strong intercellular communication. Within this network, three specific T-cell subtypes—*CD4*^+^, *CD8*^+^ and γδ T cells—are the primary producers of Thy1 ligands, mainly targeting MΦs. This differentiation in the signaling pathway suggests that T cells and MΦs have different roles within the Thy1 network. Turning to the amyloid precursor protein (APP) signaling network, MΦs are identified as the main source of APP ligands. Interestingly, APP predominantly affects DCs, pDCs, MΦs, and B/plasma cells, with dendritic cells and MΦs orchestrating and modulating the APP signaling pathway. Notably, mouse lacrimal gland cells have high levels of APP expression and release it into the tear fluid, contributing significantly to ocular surface health [150]. Taken together, these findings shed light on the diverse functions of immune cell subtypes in the lacrimal gland. 

### 3.8. Study Limitations

There are several limitations to this study that should be acknowledged. First, our observations were limited to immune cells in the one male C57BL/six mouse ELGs. Obtaining data from female mice could provide valuable insights into the gender dimorphism of lacrimal gland immune cells [151]. Additionally, due to the complexity of immune cell phenotypes, subpopulation analysis was not validated by histological immunofluorescence techniques for the majority of the immune cells. Further investigation using flow cytometry may contribute to a more comprehensive analysis of these subpopulations. Most gene expression and some immune cell recruitment to the lacrimal glands are influenced by light phase and age [7,152,153]. This study only focused on single diurnal time and age points. Finally, future research employing spatial transcriptomics could provide valuable information on the tissue localization of the aforementioned immune cell subpopulations.

### 3.9. Future Research Directions

Increasing evidence suggests that different populations of immune cells in the lacrimal gland play different roles in disease onset and progression. For example, increased numbers of Tregs have been observed in the lacrimal glands of ageing individuals [154,155]. In Sjögren’s syndrome (SS), an autoimmune disease that primarily affects the exocrine glands, including the lacrimal gland, T cells predominate early in the disease. Th1 and Th17 cells are the initiators of SS, whereas Th2 and Tfh cells become predominant as the disease progresses [156]. In addition, B cells dominate in the later stages. Furthermore, the infiltration of macrophages and monocytes in the lacrimal gland is strongly correlated with the severity of Sjögren’s syndrome [157,158]. In the mouse model of dry eye disease, cDCs in the lacrimal gland are approximately spherical and have increased migration [11]. In conclusion, the different immune cell populations in the lacrimal gland interact to coordinate the onset and development of the immune response. Based on the information obtained in this study, the lacrimal gland appears to have a specific immune microenvironment. In the future, drugs could be designed to specifically modulate the local immune response of the lacrimal gland by targeting specific receptors or signaling pathways of the immune cell populations in the lacrimal gland to treat specific ocular diseases (e.g., dry eye and ocular inflammatory diseases). In addition, a better understanding of the interactions between these immune cell populations is important to elucidate the pathogenesis of inflammatory diseases of the lacrimal gland.

## 4. Materials and Methods

### 4.1. Experimental Animals

Pathogen-free 12- to 14-week-old male C57/B6J mice were purchased from the Guangdong Medical Laboratory Animal Center (Foshan, Guangdong, China). All mice were housed in an environment with a standard 12 h light/dark cycle and appropriate temperature (23 ± 2 °C) and humidity. In addition, all animal experiments were approved by the Animal Ethics Review Committee of Jinan University (JN-A-20210303-32) and followed the guidelines provided by the Association for Research in Vision and Ophthalmology (ARVO) Statement on the Use of Animals in Ophthalmic and Vision Research.

### 4.2. Tissue Collection and Single-Cell Sample Preparation

To minimize the influence of circadian rhythm, all experimental mice were euthanized at the ZT4 time point using excess CO_2_ inhalation followed by cervical dislocation. Subsequently, we harvested bilateral ELGs, centrifuged them (4 °C, 500× *g*), and removed the supernatant, and they were then subjected to two washes with Eagle’s minimal essential medium. The ELG tissue was then swiftly sectioned into 2–3 mm fragments and subjected to incubation in a digestion solution consisting of 2 mg/mL collagenase I, 2 mg/mL collagenase IV, and 1 mg/mL DNAas I enzymes. These tissue sections were placed in C-tubes and processed in a tissue processor for 1–2 h at 37 °C.

The resultant cell suspension was collected using a 40 μm filter. To eliminate erythrocytes, we introduced erythrocyte lysis buffer (Cat# 8570396, QIAGEN, Shanghai, China) into the supernatant of the filtered cell suspension. This supernatant was then subjected to centrifugation at 220× *g* for 8 min at 4 °C, effectively removing the supernatant. We further employed the Dead Cell Removal Kit (Cat# 130-090-101, Miltenyi Biotec, Auburn, CA, USA) to eliminate dead cells and debris. Finally, the cells were thoroughly washed with phosphate-buffered saline (PBS), resuspended in an appropriate volume, and quantified using a hemocytometer.

### 4.3. Single-Cell RNA Sequencing

For the scRNA-seq experiments, we used a method that pooled two ELGs into a single sample from each male mouse, resulting in a total of four ELG samples from four young mice (*n* = 4). After dissociation of the lacrimal gland tissue, individual cells were appropriately diluted and suspended in calcium- and magnesium-free PBS supplemented with 0.04% *w/v* bovine serum albumin. Approximately 10,000 cells per sample were loaded into each channel, and the expected cell recovery rate was estimated to be 8000 cells. To generate single-cell gel beads-in-emulsions (GEMs), we introduced the single-cell suspension into the Chromium Single Cell Controller (10× Genomics, Pleasanton, CA, USA).

After GEM generation, reverse transcription reactions were performed using full-length cDNA coupled to barcodes. The emulsion was then disrupted with a recovery agent, and the cDNA was purified using DynaBeads Myone Silane beads (Thermo Fisher Scientific, Waltham, MA, USA). This cDNA underwent an amplification process consisting of 14 PCR cycles corresponding to the cDNA concentration. The amplified cDNA was then subjected to fragmentation, end repair, A-tailing, and ligation with indexed articulators for library amplification. These libraries were then sequenced on the Illumina HiSeq X Ten platform (Illumina, San Diego, CA, USA).

For each sample, we meticulously processed the Fastq files of scRNA-seq data derived from Chromium 10× libraries using Cell Ranger software (version 5.0.0, 10× Genomics, https://github.com/10XGenomics/cellranger, accessed on 11 November 2023). This involved a number of essential steps, including single-cell barcode identification, genome mapping, and quantification of unique molecular identifiers (UMIs) associated with transcripts. We then used the R package DoubletFinder (version 2.0.3) to detect and eliminate potential doublet cells within each sample to ensure the integrity of downstream analyses, and we used the Seurat package (version 4.3.0) for comprehensive analysis of the resulting filtered feature–barcode matrices.

To maintain data quality and accuracy, we implemented stringent quality control measures by excluding samples with nFeature RNA counts greater than 2500. This threshold was applied to mitigate the inclusion of samples containing an excessive number of genes, which may have resulted from doublets or multiple cells failing to form a single-cell suspension.

We further refined the data by normalizing using default parameters and the FindVariableFeatures function, which effectively filtered out highly variable genes (parameters set to selection.method = “vst” and nfeatures = 2500). These genes are used for downstream analyses such as downscaling and clustering. Dimensionality reduction analysis was then performed to select the most relevant dimensions; this involved performing principal component analysis using the RunPCA function to construct a linear dimensionality reduction of the dataset that contained most of the complexity of the dataset and evaluating the standard deviation using the ElbowPlot function. We then performed data clustering using the FindClusters function, systematically testing different resolution values from 0.4 to 1 to determine the optimal number of clusters (final resolution = 0.8). Finally, we applied nonlinear dimensionality reduction using the RunUMAP function for improved visualization.

The software resources and versions used for data analysis in this study are described above and summarized in Table S3, and all original R scripts are available at https://github.com/Qwei777/Singlecell.git, accessed on 5 January 2024. 

### 4.4. Identification of Cell Types

The initial characterization of cell types within each cluster was conducted using the SingleR package as a preliminary reference. Subsequently, differential expression analysis was primarily executed using the Seurat FindMarkers function. This allowed us to visualize cluster-specific marker genes and generate a heatmap featuring the top 10 marker genes for each cluster. Cell types were ascertained based on the marker gene expression profiles associated with each cluster, supplemented by specific identity markers for each cell type, as elucidated in the previously published literature. Additionally, to delve deeper into the heterogeneity of immune cells, we carried out further dimensionality reduction, clustering, and classification for each subgroup of immune cells.

### 4.5. Analysis of Cell-Cell Interactions and Communication

Cell-to-cell communication analysis was performed using the R package CellChat 1.6.1 (http://www.cellchat.org/, accessed on 12 November 2022). Our investigation focused on deciphering cell-to-cell interactions between different immune cell types within adult mouse ELGs. An integral aspect of this analysis was to assess the importance of cell-to-cell communication by examining ligand-receptor interactions between different cell populations. To assign biological relevance to these cell-cell communications, we adopted a probabilistic approach by assigning probability values to each interaction. A permutation test was then used to validate the significance of these interactions. Notably, we set a gene expression threshold of 0.2 as the effective cutoff point for inclusion in our analysis. This rigorous methodology ensures robust and credible insights into the intricate network of cell-to-cell interactions within the system under study.

### 4.6. Functional Signaling Pathway Enrichment Analysis

Gene Ontology (GO) enrichment analysis of the dataset was performed using the R package ClusterProfiler (version 4.2.2). The main objective of this analysis was to identify pathways that showed significant enrichment for genes associated with different immune cell populations in the study. To ensure the reliability and robustness of our findings, we implemented a strict significance threshold for enrichment. Specifically, only pathways with an adjusted *p* value < 0.05 were considered statistically significant. By adopting this rigorous approach, we aimed to increase the confidence in our results. By focusing on pathways that showed substantial enrichment, we were able to prioritize those that were more likely to have a biologically relevant role in characterizing immune cell populations.

### 4.7. Immunohistological Staining of Mouse ELGs

For immunohistological staining of mouse ELGs, a series of meticulously performed steps were followed. First, mouse ELG tissues were sectioned and thoroughly washed with 0.01 M phosphate-buffered saline (PBS) at room temperature using a shaker. The sections were then fixed through immersion in a 5% bovine serum albumin (BSA) solution for 15 min. A mixture of BSA and 0.3% Triton X-100 was then added to the samples to promote permeabilization and allowed to interact for an additional 15 min at room temperature. To facilitate specific antibody binding, slides containing the sections were immersed in a primary antibody solution. This solution, appropriately diluted in BSA supplemented with 0.3% Triton X-100, was applied and incubated overnight at a controlled temperature of 4 °C. For reference, details of the primary antibodies, including concentrations and accession numbers, are documented in Table S1. After overnight incubation with primary antibodies, the samples were thoroughly washed with PBS. They were then subjected to secondary antibody treatment for 3 h at room temperature in a light-restricted environment. Alexa Fluor secondary antibodies (Cat No: A32727, Life Technologies in Carlsbad, CA, USA) were used at a dilution of 1:500 in a solution containing 0.3% Triton X-100 in BSA. As a final step, the nuclei of the samples were stained and blocked with a blocking agent supplemented with 4′,6-diamidino-2-phenylindole (DAPI) (Cat#: 28718-90-3; Sigma-Aldrich, St. Louis, MO, USA) at a ratio of 1:500. The DAPI used in this procedure was purchased from Sigma-Aldrich, St. Louis, MO, USA. This extensive procedure was carried out meticulously to ensure accurate and reliable immunohistological staining of mouse ELGs.

### 4.8. Statistical Analysis

Statistical analyses were performed using R software 4.1.3 (http://www.r-project.org, accessed on 10 March 2022). Seurat’s standard nonparametric Wilcoxon rank sum test was used, and *p* values were corrected using Bonferroni correction across all features in the dataset. Data are expressed as the mean and standard error of the mean (SEM). Only *p* values < 0.05 were considered statistically significant.

## 5. Conclusions

In conclusion, our study provides a comprehensive understanding of the immune system within the ELGs of normal mice. These data provide new insights regarding not only the structural and functional aspects of the lacrimal gland but also the pathogenesis of inflammatory diseases associated with the lacrimal gland; these include conditions such as dry eye syndrome, Sjogren’s syndrome, and various autoimmune diseases. In addition, our findings may contribute to the development of novel therapeutic strategies for these conditions.

## Data Availability

All raw sequencing data reported have been deposited at this accession URL: https://singlecell.broadinstitute.org/single_cell/study/SCP2459. All data were analyzed with standard programs and packages; original code has been deposited at https://github.com/Qwei777/Singlecell.git, accessed on 5 January 2024.

## Supplementary Materials

The following supporting information can be downloaded at: https://www.mdpi.com/article/10.3390/ijms25021208/s1.

## Abbreviations

APP	amyloid precursor protein
BP	biological process
cDC	conventional dendritic cell
CCL	CC-chemokine ligand
CCR	CC-chemokine receptor
CXCL	chemokine (C-X-C motif) ligand
CXCR	chemokine (C-X-C motif) receptor
DCs	dendritic cells
ELG	extraorbital lacrimal gland
GO	gene ontology
IFN	interferon
IgA	immunoglobulin A
ILCs	innate lymphoid cells
Jak-STAT	the Janus kinase/signal transducers and activators of transcription
MCs	mast cells
mDCs	migratory dendritic cells
NKs	natural killers
pDCs	plasmacytoid dendritic cells
Thy1	thymus cell antigen 1
Tregs	regulatory T cells
TRM	tissue-resident memory
UMAP	uniform manifold approximation and projection
