# Supplementary material for: Exploring Immune Cell Diversity in the Lacrimal Glands of Healthy Mice: A Single-Cell RNA-Sequencing Atlas
Source: Int J Mol Sci. 2024 Jan 19;25(2):1208. doi: 10.3390/ijms25021208 (PMC10816500; doi:10.3390/ijms25021208)
Supplement: Supplementary file 1 [file ijms-25-01208-s001.zip › ijms-2766697 - Supplementary Figures.pdf]

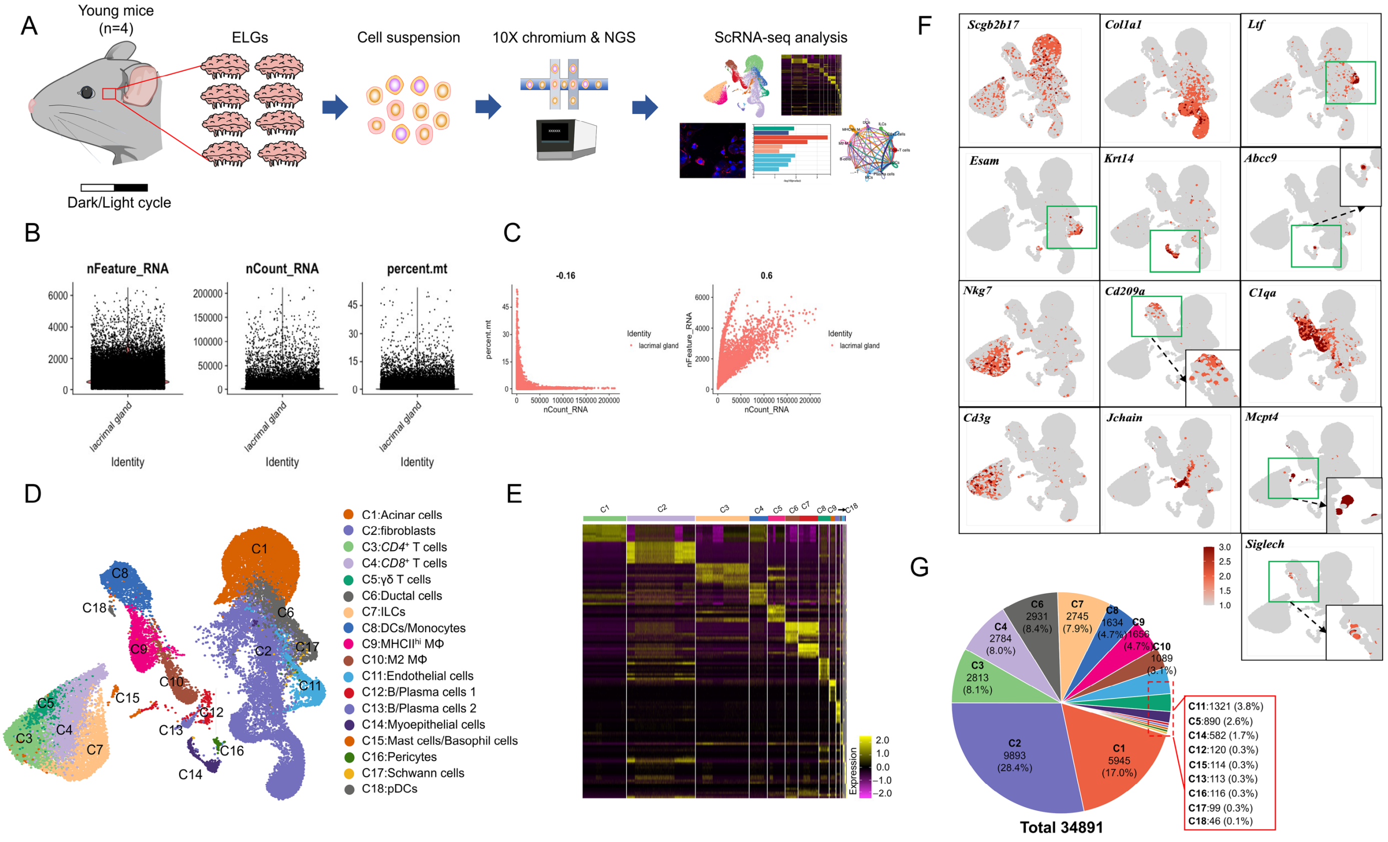

Figure S1. Mouse ELG scRNA-seq quality control and initial cell identification. Four biological replicates (eight lacrimal gland samples) were collected from adult mice to obtain a sufficient number of single cells. The samples were transported to the laboratory within eight hours and dissociated into single cells. The scRNA-seq analysis was performed using the 10X Genomics Chromium platform (**Figure S1A**). Cell Ranger was employed for quantitative quality control, including demultiplexing, read alignment, and identification of empty droplets, to ensure a high-quality cell count distribution ranging from 9,245 to 15,122. Standard quality control measures were then applied to exclude doublets, multiplets, and apoptotic cells. A total of 34,891 high-quality single cells were obtained for further analysis. Seurat (version 4.3.0) was used to cluster and group the scRNA-seq data. Based on the differentially expressed gene (DEG) signatures, we identified relatively specific genes that were highly enriched in each cell cluster (**Table S2**). (**A**) Schematic workflow of scRNA-seq in this study. (**B, C**) Quality control was performed to select cells for further analysis. Scatter plots were used to display nFeature_RNA (number of genes detected in each cell), nCount_RNA (number of molecules detected in each cell), percent.mt (mitochondrial DNA ratio) and percent mitochondrial genomes vs. counts and features vs. counts. (**D**) Single-cell transcriptomic data from a total of 34,891 cells were obtained from eight lacrimal gland tissues from four male C57BL/6J mice. A UMAP plot of the lacrimal gland cells was generated, and 18 cell clusters were identified using the Seurat package v4.1.3. (**E**) Heatmap of Z scores for scaled expression values of DEGs for each cluster. Colors are based on the natural logarithm of normalized RNA expression. (**F**) UMAP feature plots were generated to highlight highly expressed genes in each cell cluster. (**G**) The pie chart illustrates the number of cells in each cell subpopulation and their respective proportion of the total number of cells.

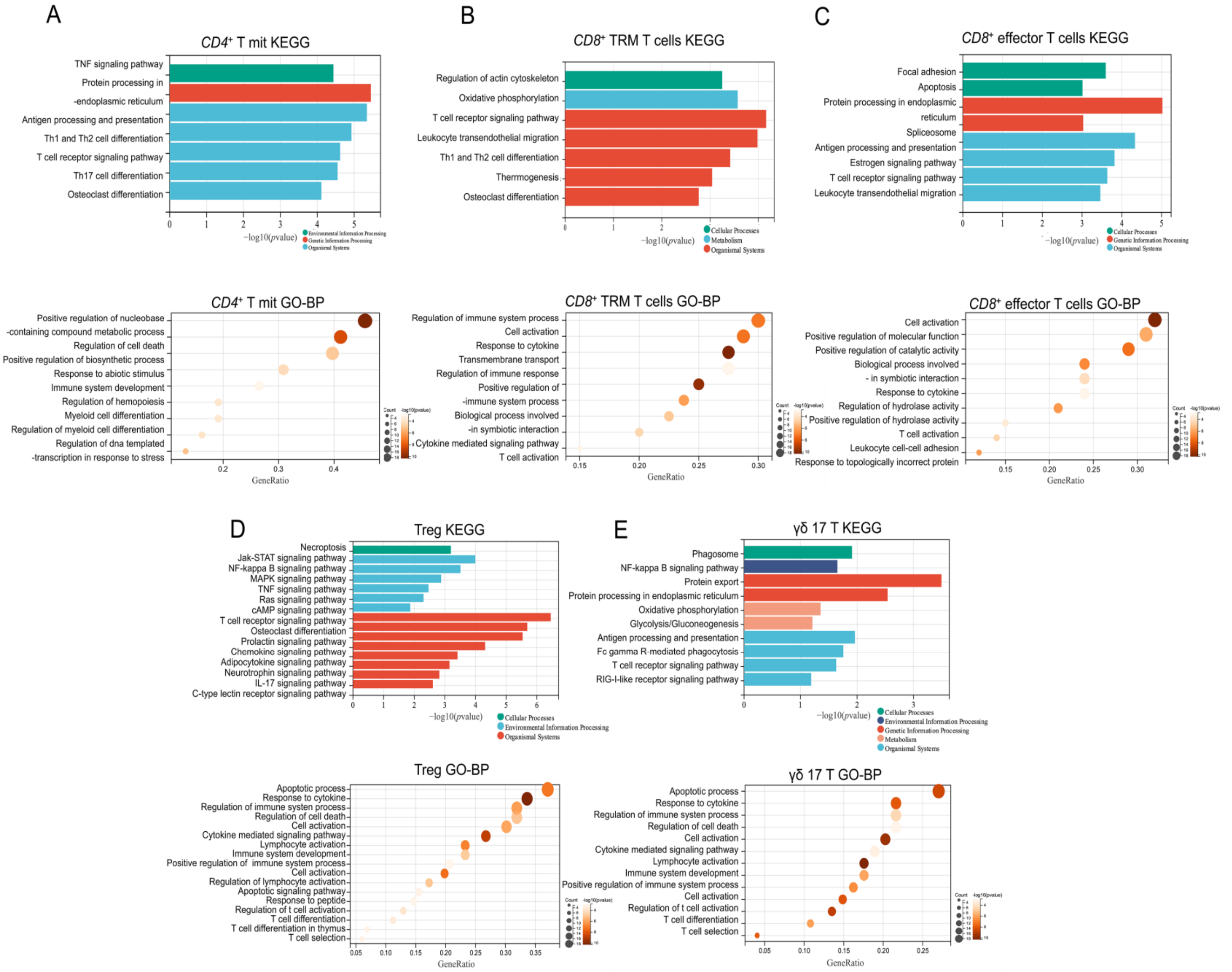

Figure S2. KEGG and GO functional enrichment analysis of T-cell subsets. (**A**) Bar graphs showing the signaling pathways enriched by KEGG analysis in CD4+ T_mit cell populations; bubble plot illustrating the biological processes analyzed by GO in these cell populations. (**B**) Bar graphs showing the signaling pathways enriched by KEGG analysis in CD8+ TRM cell populations; bubble plot illustrating the biological processes analyzed by GO in these cell populations. (**C**) Bar graphs showing the signaling pathways enriched by KEGG analysis in CD8+ effector cell populations; bubble plot illustrating the biological processes analyzed by GO in these cell populations. (**D**) Bar graphs showing the signaling pathways enriched by KEGG analysis in Treg cell populations; bubble plot illustrating the biological processes analyzed by GO in these cell populations. (**E**) Bar graphs showing the signaling pathways enriched by KEGG analysis in γδ 17 T-cell populations; bubble plot illustrating the biological processes analyzed by GO in these cell populations.

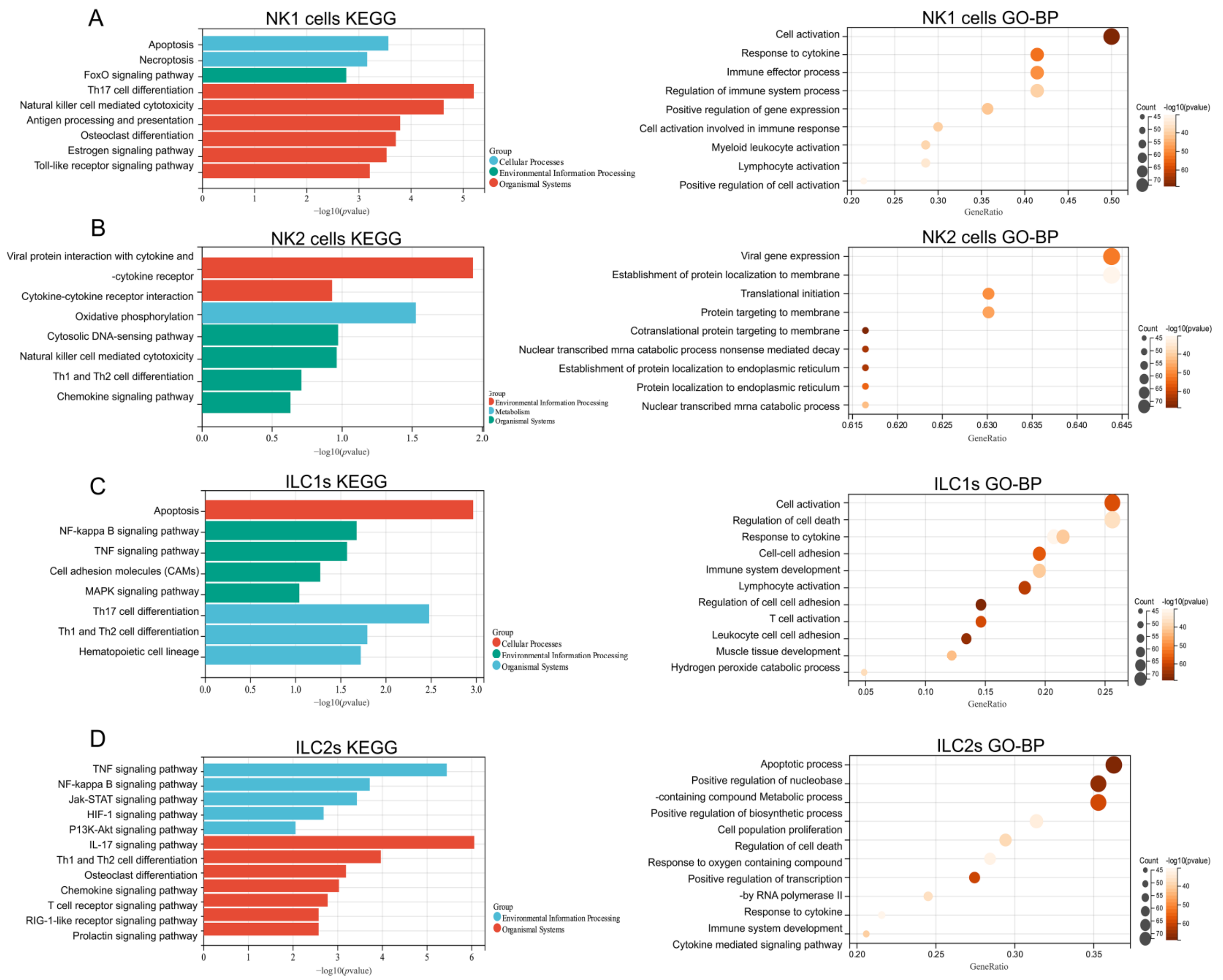

Figure S3. KEGG and GO-BP analyses of genes expressed in each ILC subpopulation. (**A**) Bar graphs depict enriched KEGG pathway analysis results for the NK1 cell cluster, while bubble plots illustrate the biological processes from the GO analysis of this cell group. (**B**) Bar graphs display enriched KEGG pathway analysis results for the NK2 cell cluster, and bubble plots represent the biological processes from the GO analysis of this cell group. (**C**) Bar graphs showcase enriched KEGG pathway analysis results for the ILC1 cell cluster, with bubble plots visualizing the biological processes from the GO analysis of this cell group. (**D**) Bar graphs exhibit enriched KEGG pathway analysis results for the ILC2 cell cluster, while bubble plots display the biological processes from the GO analysis of this cell group.

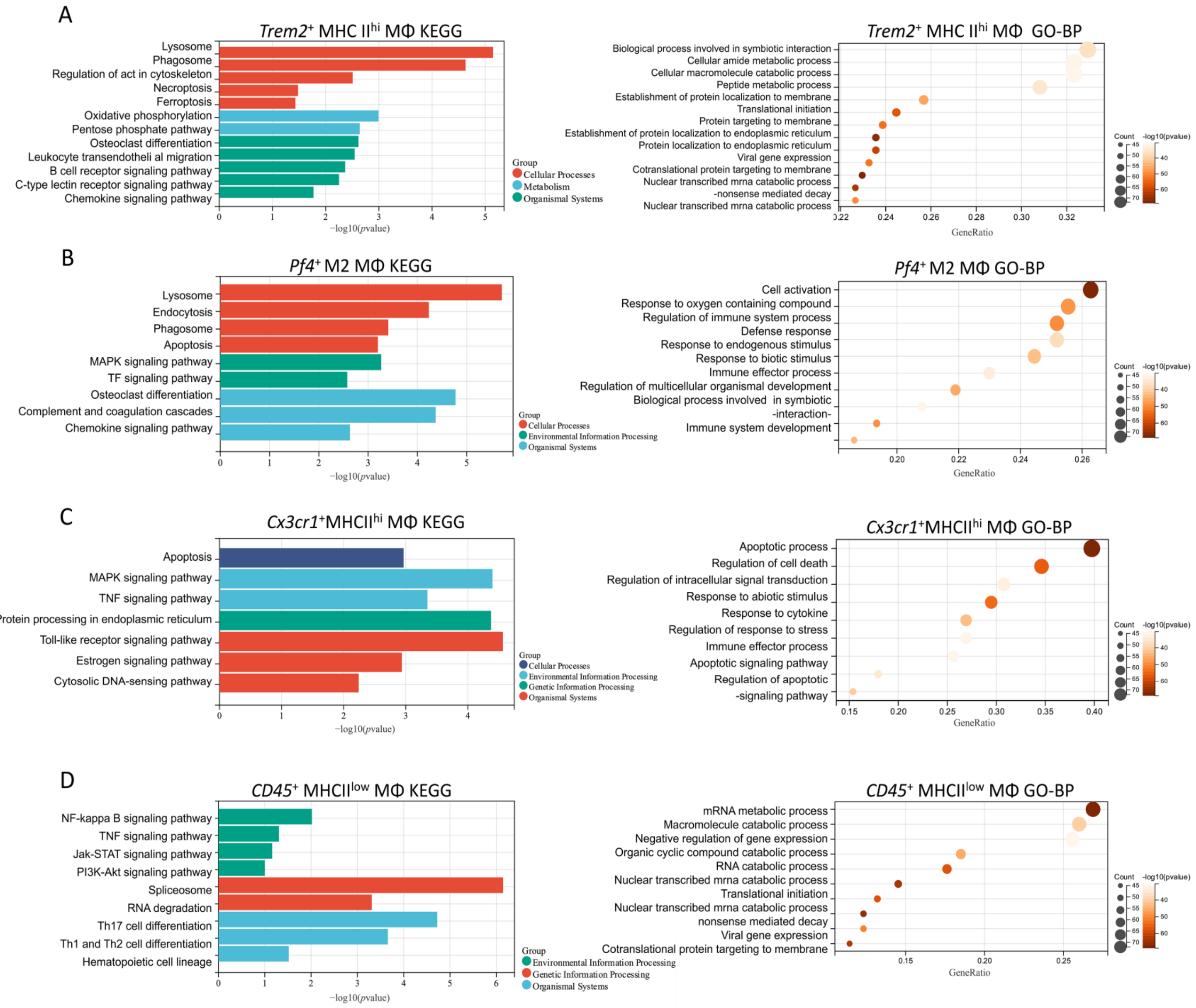

Figure S4. KEGG and GO analysis of different macrophage subpopulations. (**A**) Bar graphs showing the enriched KEGG pathway analysis results for the *Trem2*+ MHC II^{hi} macrophage subpopulation, while the bubble plot illustrates the biological processes from the GO analysis of this cell group. (**B**) Bar graphs showing the enriched KEGG pathway analysis results for the *Pf4*+ M2 MΦ subpopulation, and bubble plot illustrating biological processes from the GO analysis of this cell group. (**C**) Bar graphs showing the KEGG pathway analysis results for the *Cx3cr1*+ MHCII^{hi} MΦ subpopulation, with a bubble plot illustrating the biological processes from the GO analysis of this cell group. (**D**) Bar graphs showing the KEGG pathway analysis results for the *CD45*+ MHCII^{low} subset of MΦs, with a bubble plot illustrating the biological processes from the GO analysis of this cell group.

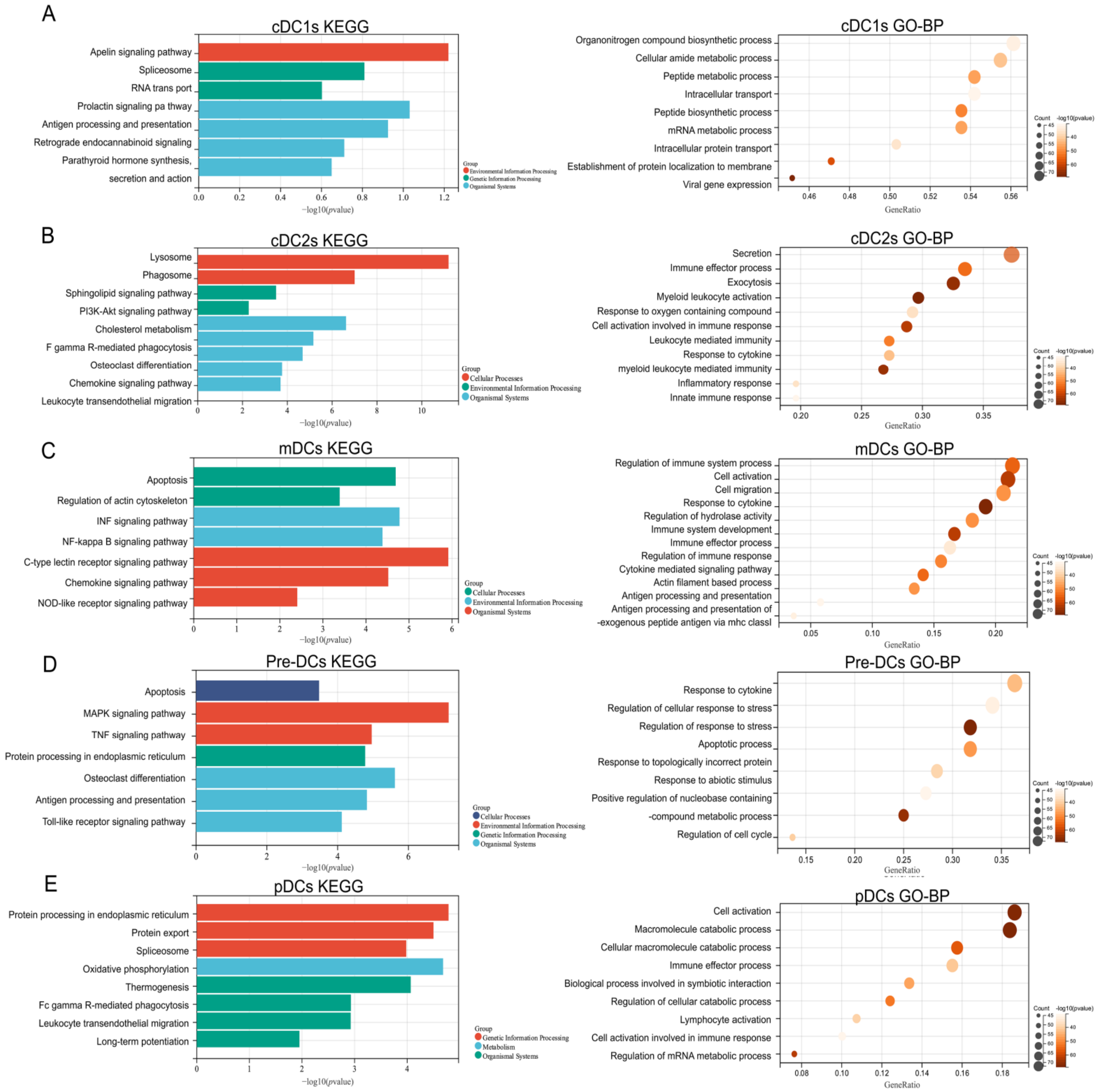

Figure S5. KEGG and GO analysis of different DC subpopulations. (**A**) Bar chart displaying enriched KEGG pathways in the cDC1 subpopulation; bubble plot presenting GO analysis of biological processes for the cell group. (**B**) Bar chart displaying enriched KEGG pathways in the cDC2 subpopulation; bubble plot showing GO analysis of biological processes for the cell group. (**C**) Bar chart displaying enriched KEGG pathways in the mDC cell group; bubble plot illustrating GO analysis of biological processes for the cell group. (**D**) Bar chart displaying enriched KEGG pathways in the pre-DC cell group; bubble plot representing GO analysis of biological processes for the cell group. (**E**) Bar chart displaying enriched KEGG pathways in the *Ccr9*+ pDC cell group; bubble plot demonstrating GO analysis of biological processes for the cell group.

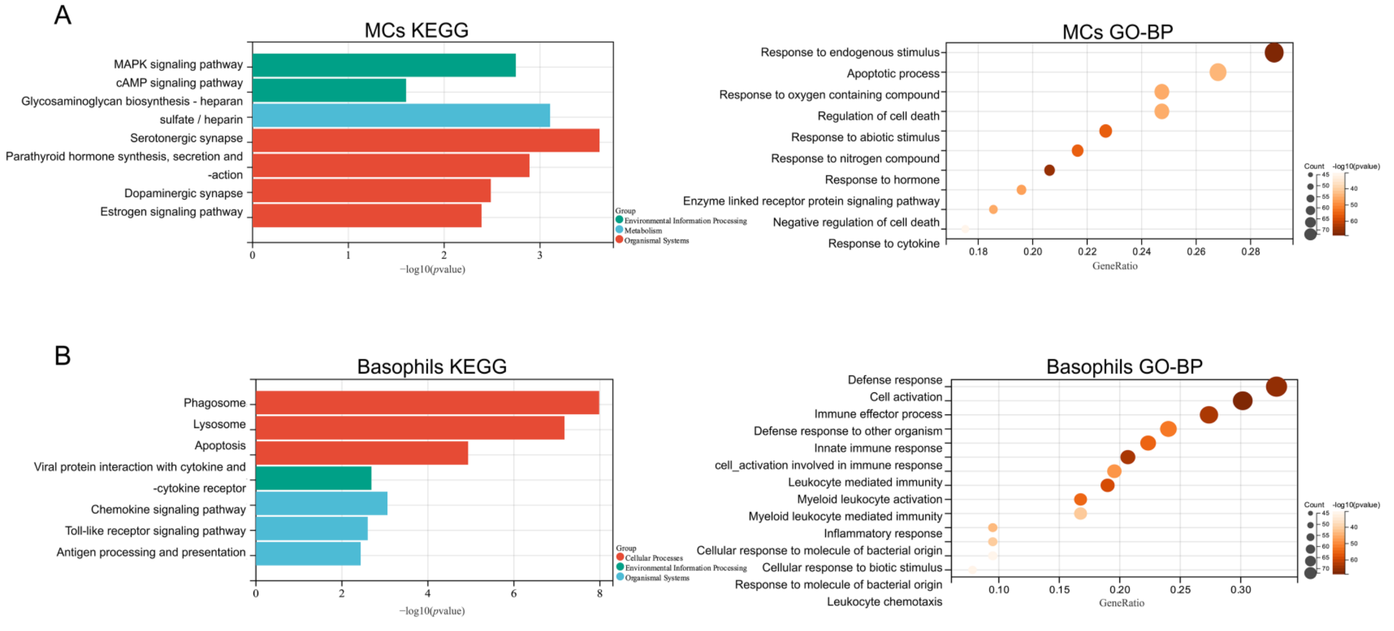

Figure S6. KEGG and GO analysis of the genes expressed in MCs and basophils. (**A**) The bar graph shows the enriched pathways identified by KEGG analysis in the MC cell population. The bubble plot illustrates the biological processes enriched in the cell population as determined by GO analysis. (**B**) The bar graph depicts the enriched pathways identified by KEGG analysis in the basophil cell population. The bubble plot shows the biological processes enriched in the cell population as determined by GO analysis.

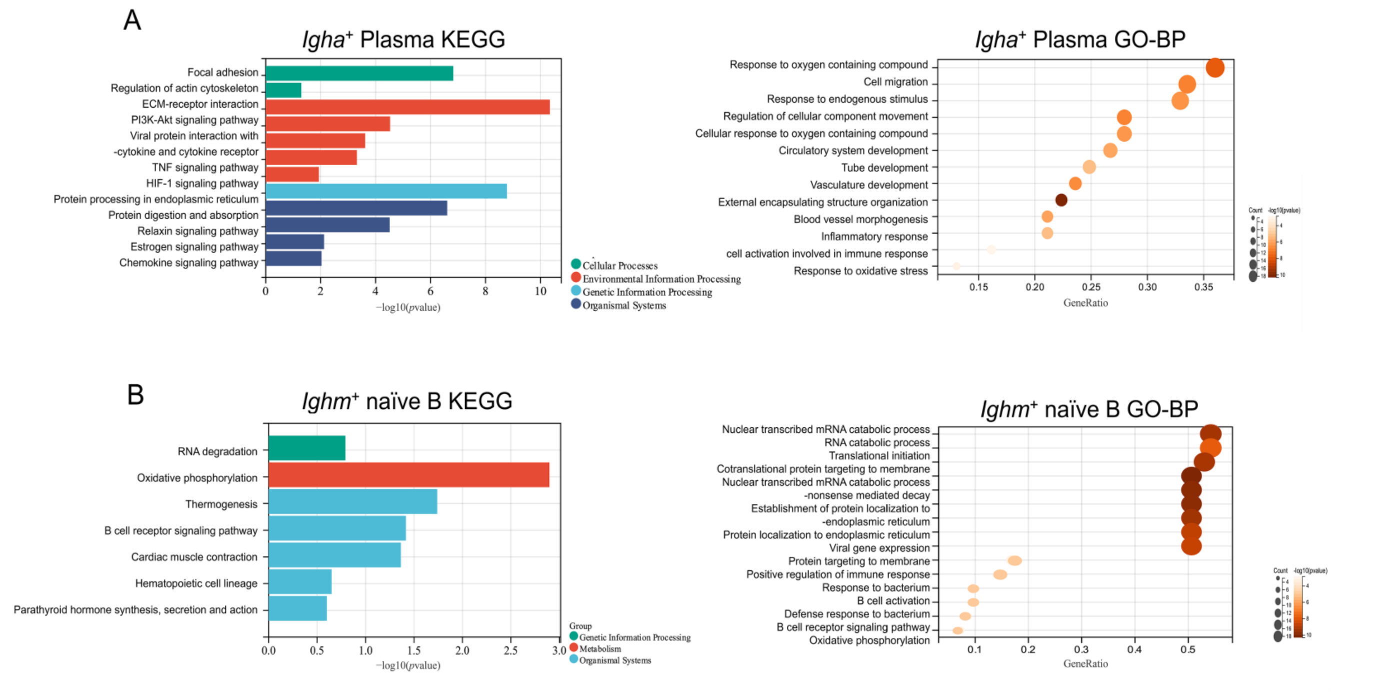

Figure S7. KEGG and GO analysis of genes expressed in the B-cell/plasma cells cluster. (**A**) The bar graph and bubble plot depict the functional KEGG analysis and GO analysis of biological processes for the *Igha*+ plasma cell population. (**B**) The bar graph and bubble plot represent the functional KEGG analysis and GO analysis of biological processes for the *Ighm*+ naive B- cell population.
